# Efficient Approaches to the Design of Six-Membered Polyazocyclic Compounds—Part 2: Nonaromatic Frameworks

**DOI:** 10.3390/molecules30193911

**Published:** 2025-09-28

**Authors:** Yuliya Yu. Titova, Elena A. Gyrgenova, Andrey V. Ivanov

**Affiliations:** A.E. Favorsky Irkutsk Institute of Chemistry, The Siberian Branch of the Russian Academy of Sciences, Favorsky Str. 1, 664033 Irkutsk, Russia; e.gyrgenova@irioch.irk.ru (E.A.G.); ivanov@irioch.irk.ru (A.V.I.)

**Keywords:** hydrogenation, saturation, dearomatic binding, six-membered polyazo heterocycles

## Abstract

This review summarizes and compares literature data on the main strategies for the synthesis of saturated and/or partially saturated analogs of six-membered polyazoaromatic heterocycles. With a few exceptions, this data was published within the last 15–20 years. These strategies include, first of all, hydrogenation, which can be carried out in a classical manner (i.e., in the presence of a catalyst and molecular hydrogen) or via hydrogen transfer techniques. Other approaches comprise saturation of aromatic frameworks, which can be achieved using compounds other than hydrogen, such as hydroboration and dearomatic binding, or the creation of saturated or partially saturated polyazo heterocycles through the coupling of two or more molecules. Each of the above strategies has certain advantages and serious shortcomings and limitations. Despite the apparent methodological difficulties, it is demonstrated that the described approaches can sometimes give impressive results, including the production of optically pure products under relatively mild conditions.

## 1. Introduction

**Figure 1:**
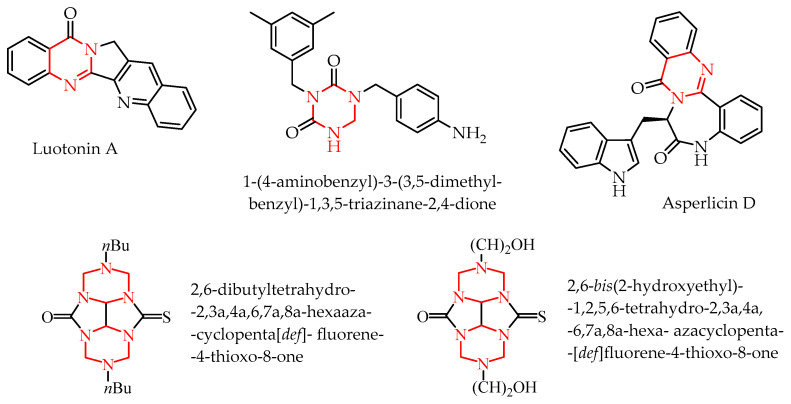
Examples of registered drugs containing saturated or partially saturated analogs of polyazoaromatic heterocycles (the target polyazole nonaromatic cores are highlighted in red).

Saturated or partially saturated analogs of polyazoaromatic heterocycles are important structural motifs of biologically active molecules and/or pharmaceuticals (Figure 1) [1,2,3,4,5,6,7,8,9,10,11,12,13,14,15]. For example, Luotonin A exhibits cytotoxicity against the murine leukemia cell line P-388, as well as possesses antiviral and fungicidal activities [16]. Asperlicin D has antagonistic activity against cholecystokinin [17]; 1-(4-aminobenzyl)-3-(3,5-dimethylbenzyl)-1,3,5-triazine-2,4-dione is active against human immunodeficiency virus (anti-HIV-1) [18]; 2,6-dibutyltetrahydro-2,3a,4a,6,7a,8a-hexaazacyclopenta[*def*]-fluorene-4-thioxo-8-one and its analogs have antimicrobial activities [19] etc.

Although hydrogenation or dearomatization of polyazocyclic compounds is considered to be one of the most convenient methods for the synthesis of their saturated analogs, this strategy is rather difficult to implement [20]. This phenomenon can be attributed to a number of factors, including the high aromaticity and stability of cyclic π-electronic systems, as well as the presence of several strong coordinating nitrogen atoms, both in the starting compounds and the molecules of possible products, which contribute to the catalyst poisoning [21]. Therefore, despite over a century of research in this area, the development of methodologies that can selectively convert *N*-heteroarene rings into the corresponding saturated or partially saturated cyclic *N*-containing skeletons under mild conditions remains an extremely challenging and urgent task [22].

On the other hand, disruption of the cyclic π-system of a heteroaromatic compound usually results in the formation of at least one sp^3^-hybridized carbon atom that can become a chiral center. This adds diversity and complexity to the now chiral-saturated or partially saturated heterocyclic skeleton, enabling the creation of structures that closely resemble natural compounds. However, most current strategies for saturation of different heterocycles are based on heteroaromatic compounds with only one heteroatom [22,23,24,25].

**Figure 2:**
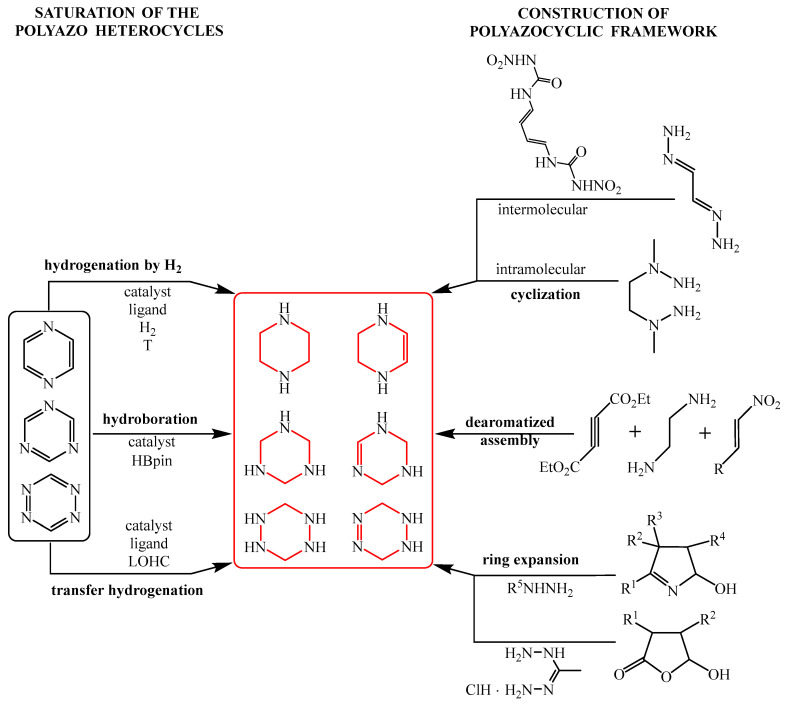
General methods for the synthesis of nonaromatic polyazocyclic compounds.

A comprehensive review of the literature indicates that there are three main methodologies for the preparation of saturated polyazocyclic compounds (see Figure 2). The first one is the hydrogenation of the corresponding unsaturated analogs, which is accomplished through the addition of one or more hydrogen molecules. Moreover, the hydrogenation of frameworks containing two nitrogen atoms can be achieved in two ways: (1) classical hydrogenation in the presence of molecular hydrogen and a catalyst, whereby often one nitrogen atom is preliminarily blocked by alkylation and the more basic secondary nitrogen atom is transformed into a salt by the action of an acid obtained in situ. The chiral version of these techniques is realized in the presence of special ligands, whose physicochemical properties allow them to retain chirality under harsh conditions (moderate to high pressures, a wide temperature range, and long reaction times) [23,26,27,28,29,30]; (2) so-called transfer hydrogenation, where the hydrogen source is a substance that is transformed during hydrogenation into a new molecule, which is ideally unable to react with either the original substrate or the target product(s). This method does not require a change in pressure; however, it is imperative to exercise caution when selecting the composition of catalytic systems [31]. To date, no examples of hydrogenation of molecules containing triazine and tetrazine fragments using these methods have been found.

The second methodology is saturation, i.e., the addition of one or more molecules to the *N*-arene ring. The most well-developed examples described in the literature are hydroboration processes [32,33].

The third methodology involves the construction of polyazocyclic compounds, which is carried out, e.g., through the formation of the corresponding saturated *N*-heterocyclic framework via the binding of two or more molecules and ring expansion reactions, as well as intramolecular and intermolecular cyclizations [10,34,35,36,37] (see Figure 2). This process can be realized under a wide range of conditions, which are largely determined by the nature of the target products.

In spite of the fact that some attention has been paid to the synthesis of saturated polyazocyclic frameworks, it is evident that this field of research is still in its nascent stage. Moreover, the question of the scientific selection of an effective methodology for a particular model substrate has not yet been addressed. It should be underlined that the development of new techniques is contingent upon a comprehensive understanding of the scientific literature, particularly that which has been published within the last two decades. This review will therefore summarize literature data from the last twenty years describing various experimental protocols for the synthesis of saturated and/or partially saturated nonaromatic polyazocyclic frameworks, using pyridazine, pyrimidine, pyrazine, and tri- and tetra-azo fragments as examples. The information is presented in accordance with the increasing number of nitrogen atoms in the six-membered ring. First, hydrogenation techniques will be surveyed, followed by a discussion of saturation, predominantly hydroboration methods, and finally, dearomatization possibilities.

## 2. Saturation of the Six-Membered Polyazo Heterocycles

### 2.1. Methodology of Classical Hydrogenation

#### 2.1.1. Pyridazine Moiety

**Scheme 1:**
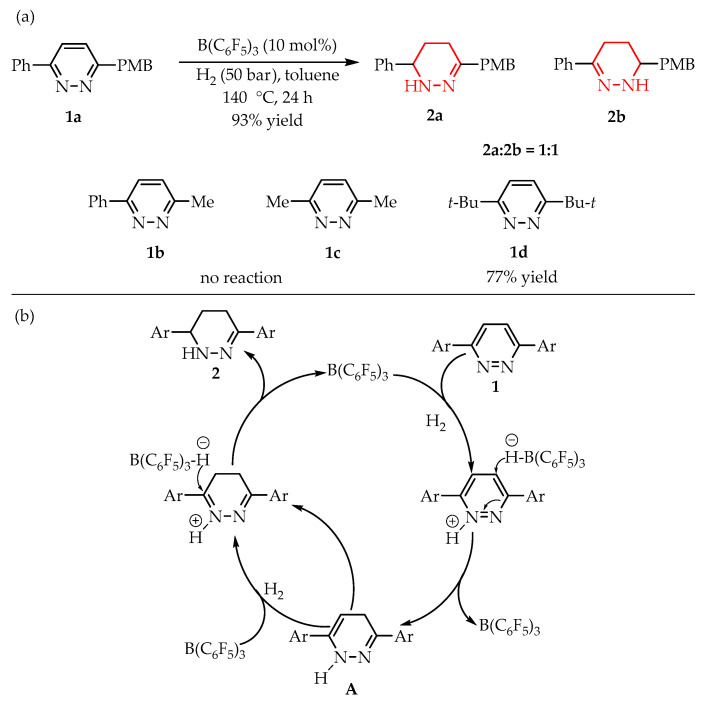
(**a**) Borane-catalyzed hydrogenation of 3,6-disubstituted pyridazines; (**b**) a plausible catalytic cycle, according to [38].

Du et al. [38] reported the first transition or rare-earth metal-free hydrogenation of 3,6-diarylpyridazines **1** using B(C_6_F_5_)_3_ as a catalyst (Scheme 1a). Various derivatives of 1,4,5,6-tetrahydropyridazine **2** were obtained in 77–95% yields. A tentative reaction mechanism was proposed (Scheme 1b).

It was suggested that H_2_ is cleaved in the presence of B(C_6_F_5_)_3_ and **1** to afford compound **A** (Scheme 1b). The second H_2_ molecule is cleaved in the presence of B(C_6_F_5_)_3_ and A, delivering product **2.** It was noted that the reason for the hydrogenation stopping at this stage is not yet clear.

Although the authors [38] claim to have successfully realized their method for the first time using commercially available B(C_6_F_5_)_3_ as a catalyst, the high price of B(C_6_F_5_)_3_ and the relatively elevated hydrogen pressure required (50 bar) are significant drawbacks of this work.

#### 2.1.2. Pyrimidine Moiety

**Scheme 2:**
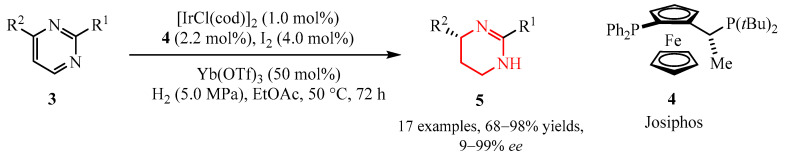
Catalytic asymmetric hydrogenation of 4-methyl-2-phenylpyrimidine, according to [39]. R^1^ = Ph, 4-CF_3_C_6_H_4_, 4-MeOC_6_H_4_, 2-FC_6_H_4_, 2-ClC_6_H_4_, 2-MeC_6_H_4_, 2-MeOC_6_H_4_, Me, NMe_2_; R^2^ = Me, Et, *c*-Hex, *t*-Bu, Ph, 4-CF_3_C_6_H_4_, 4-MeOC_6_H_4_, CH_2_OAc, CF_3_, CO_2_Et, Ph.

The asymmetric hydrogenation of pyrimidines **3** with high enantioselectivity (up to 99% *ee*) in the presence of an iridium catalyst consisting of [IrCl(cod)]_2_, a ferrocene-containing chiral diphosphine ligand **4** (Josiphos), iodine, and Yb(OTf)_3_ (where cod = 1,5-cyclooctadiene) was reported in [39] (see Scheme 2). It was shown that ytterbium triflate is crucial for both achieving high enantioselectivity and activating the heteroarene substrate.

**Scheme 3:**
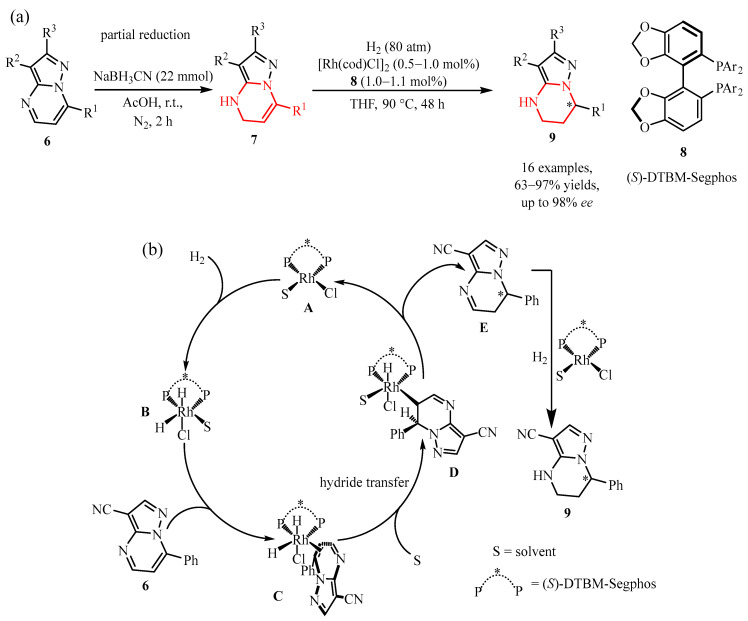
(**a**) Rh-catalyzed asymmetric hydrogenation of 4,5-dihydropyr-azolo [1,5-*a*]pyrimidine (* symbol denotes an optically active center); (**b**) proposed catalytic cycle, according to [40]. R^1^ = Ph, 3-Me-C_6_H_4_, 3-MeO-C_6_H_4_, 4-*t*Bu-C_6_H_4_, 3-X-C_6_H_4_ (X = Cl, Br, F), 4-X-C_6_H_4_ (X = Cl, Br, F) and others; R^2^ = NC, EtOOC; R^3^ = Ph, 2-Me-C_6_H_4_, 2-MeO-C_6_H_4_, 3-Me-C_6_H_4_, 4-Me-C_6_H_4_, 4-MeO-C_6_H_4_ and others; R^3^ = H; and Ar = 3,5-*t*Bu-4-MeO-C_6_H_2_; *—optically active center.

In 2023, Hao et al. [40] implemented the rhodium-catalyzed reductive dearomatization of 7-substituted pyrazolo[1,5-α]pyrimidines (**6**) to chiral 4,5,6,7-tetrahydropyrazolo[1,5-α]pyrimidines (**9**) with excellent enantioselectivity (ee up to 98%) (Scheme 3a). A plausible reaction mechanism was proposed (Scheme 3b).

Based on the results of DFT calculations and experiments involving deuterium, it was postulated [40] that rhodium complex **A** (Scheme 3b) first undergoes oxidative addition with H_2_ to form compound **B**. Then, substrate **6** coordinates with **B** to give compound **C**, followed by hydride transfer to compound **D**, accompanied by the formation of intermediate compound **E**, and complex **A** is regenerated by reductive elimination of compound **D**. Finally, intermediate compound **E** can further undergo hydrogenation to afford the final product **9**.

**Scheme 4:**
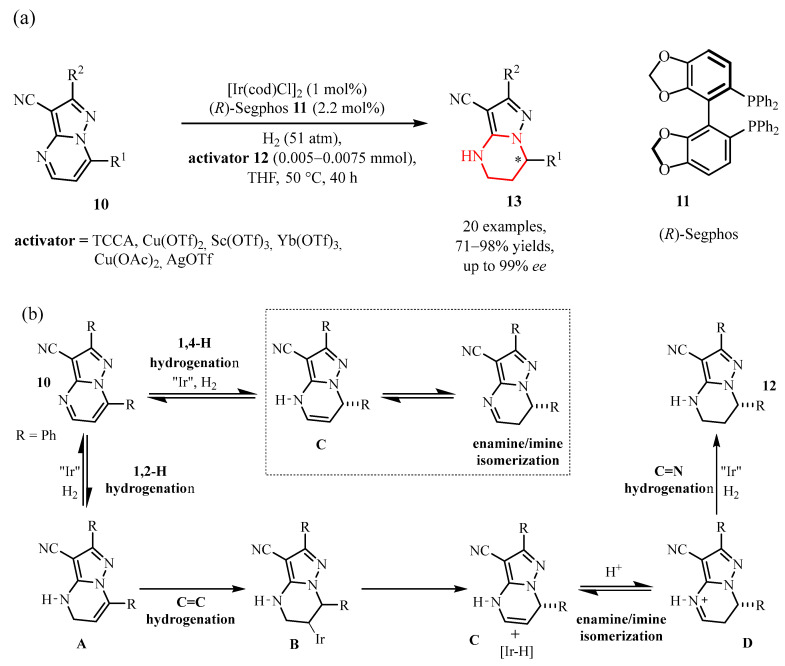
(**a**) Ir-catalyzed asymmetric hydrogenation of 2,7-diphenylpyrazolo[1,5-*a*]pyrimidine-3-carbonitrile; (**b**) proposed reaction pathway, according to [41]. R^1^ = Ph, 2-Me-C_6_H_4_, 1-Me-C_6_H_4_, 3-X-C_6_H_4_ (X = Me, Br, SMe, CF_3_) and others; R^2^ = Me, Ph, 2-Me-C_6_H_4_, 3-X-C_6_H_4_ (X = Me, MeO, CF_3_) and others; *—optically active center.

Zhou et al. [41] described an efficient iridium-catalyzed asymmetric hydrogenation of pyrazolo[1,5-α]pyrimidines **10** (*ee* up to 99%) (Scheme 4a). Trichloroisocyanuric acid, Cu(OTf)_2_, Sc(OTf)_3_, Yb(OTf)_3_, and AgOTf were employed as activating agents (**12**). The synthesis was carried out on a decagram scale. The potential and promise of this approach were highlighted. In addition, a possible reaction mechanism was presented (Scheme 4b).

The process described in Scheme 4b begins with 1,2-hydride addition to form a semihydrogenated olefin intermediate **A**. This reaction is reversible since **A** can be dehydrogenated in the presence of an iridium catalyst. The C6–C7 double bond of **A** is then incorporated into the Ir–H bond to form **B**. Next, β-hydride elimination affords an enamine intermediate **C**, which can be isolated. Then, acid-catalyzed isomerization of the enamine/imine produces an iminium-containing intermediate **D**, followed by hydrogenation to give the desired product **12**.

**Scheme 5:**
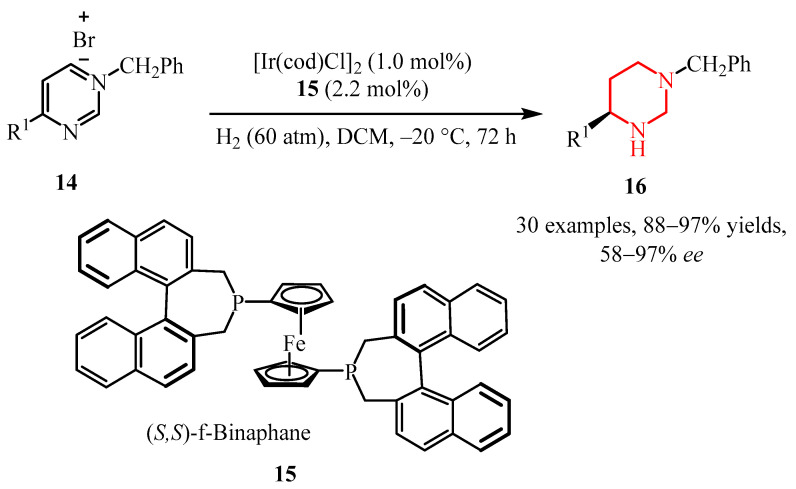
Ir-catalyzed asymmetric hydrogenation of the *N*-benzyl-4-substituted pyrimidinium salts, according to [42]. R^1^ = Ph, CH_3_-C_6_H_4_, *i*-Pr-C_6_H_4_, CH_3_O-C_6_H_4_, O_2_N-C_6_H_4_, CF_3_-C_6_H_4_, CN-C_6_H_4_, (CH_3_)_2_C_6_H_3_, X-C_6_H_4_ (X = Cl, Br, F).

A highly enantioselective method for the complete hydrogenation of pyrimidinium salts **14** using the Ir/(*S*,*S*)-f-Binaphane complex as a catalyst was disclosed in [42]. This approach provides easy access to a variety of fully saturated chiral hexahydropyrimidines **16**, which are ubiquitous in numerous bioactive molecules. The reaction proceeds under mild conditions to afford the products in high yields and enantioselectivity (Scheme 5). It was noted that the implementation of this process in continuous flow mode further extended its practical relevance.

Interestingly, in addition to the possibility of carrying out the transformations described in Scheme 5 at −20 °C, the authors presented data on performing this reaction in a flow at 50–80 °C, with R^1^ = Ph. The optimal ligand was selected at room temperature. Furthermore, a gram-scale experiment revealed that the reaction time was 168 h, rather than 72 h, as indicated in Scheme 5.

**Scheme 6:**
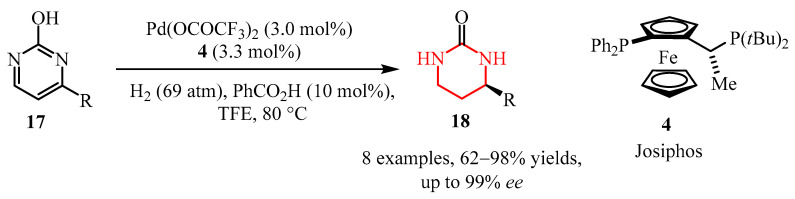
Pd-catalyzed asymmetric hydrogenation of 2-hydroxypyrimidines, according to [43]. R = Ph, CH_3_-C_6_H_4_, (CH_3_)_2_C_6_H_3_, X-C_6_H_4_ (X = Cl, Br, F), *n*-Pr-C_6_H_4_, MeO-C_6_H_4_, CF_3_-C_6_H_4_, (OCH_3_)_2_C_6_H_3_.

An efficient method for palladium-catalyzed asymmetric hydrogenation of 2-hydroxypyrimidines **17** with *ee* up to 99% was elaborated in [43] (Scheme 6). Unfortunately, this approach requires not only high hydrogen pressure but also a rather complex multicomponent catalytic system, which can be considered a notable drawback.

Despite the possibility of obtaining compounds that cannot be obtained by other means, all the methods presented in this section have a significant drawback: the need to work at high hydrogen pressure (over 50 bar). This requires specialized equipment and adherence to strict safety regulations.

#### 2.1.3. Pyrazine Moiety

**Scheme 7:**
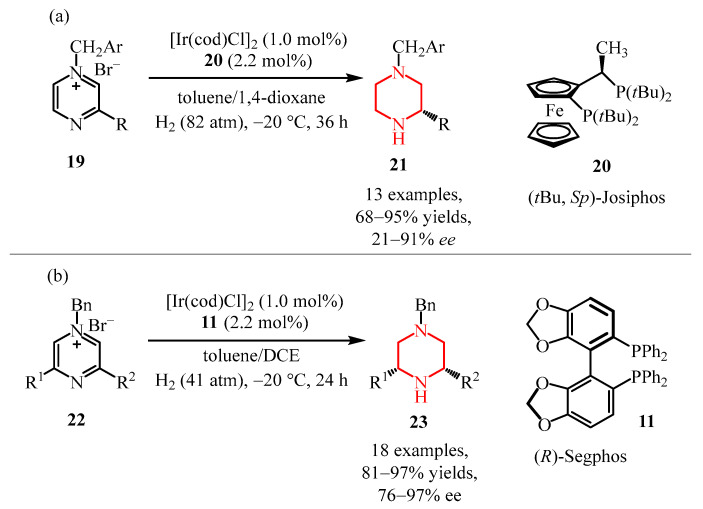
Ir-catalyzed hydrogenation of (**a**) 3-substituted and (**b**) 3,5-disubstituted pyrazinium salts, according to [44]. Ar = Ph, 2-*i*-PrO_2_CC_6_H_4_; R = Ph, 3-MeC_6_H_4_, 4-MeC_6_H_4_, 3,5-Me_2_C_6_H_4_, 3-MeOC_6_H_4_, X-C_6_H_4_ (X = Cl, Br, F), 4-CF_3_C_6_H_4_, 4-PhC_6_H_4_, and others; R^1^ = Me, Et, *n*-Pr, *n*-Bu, *i*-Bu, cyclopropyl; and R^2^ = Ph, 3-MeC_6_H_4_, 4-MeC_6_H_4_, 3,5-Me_2_C_6_H_4_, 3-MeOC_6_H_4_, 4-MeOC_6_H_4_, 4-BuOC_6_H_4_, X-C_6_H_4_ (X = Cl, F), 4-CF_3_C_6_H_4_, 4-C_6_H_5_-C_6_H_4_, and others.

Zhou et al. [44] proposed a strategy in which one nitrogen of the product was blocked by alkylation, and the more basic secondary nitrogen of piperazine formed a salt with the acid obtained in situ (Scheme 7). The reaction was accomplished in the presence of catalytic systems based on the iridium complex [Ir(cod)Cl]_2_ and chiral ligands, such as (*t*Bu, *Sp*)-Josiphos (**20**) (Scheme 7a) and (*R*)-Segphos (**11**) (Scheme 7b).

**Scheme 8:**
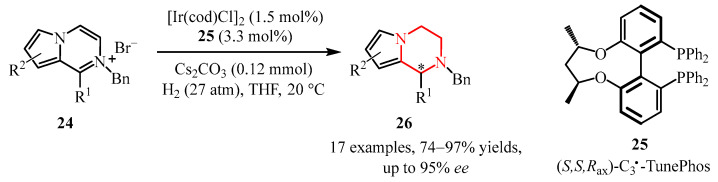
Ir-catalyzed asymmetric hydrogenation of pyrrolo [1,2-*a*]pyrazinium salts, according to [26]. R^1^ = Ph, 3-MeC_6_H_4_, 3-MeOC_6_H_4_, 4-MeC_6_H_4_, 4-*t*-BuC_6_H_4_, 4-FC_6_H_4_, 3-ClC_6_H_4_, 4-ClC_6_H_4_, 3,4-F_2_C_6_H_3_, 4-CF_3_C_6_H_4_, 4-NCC_6_H_4_, 4-EtO_2_CC_6_H_4_, 4- C_6_H_5_-C_6_H_4_, 2-naphthyl, Me, Ph; R^2^ = H, 6-Me, and others; *—optically active center.

It was shown that a highly enantioselective iridium-catalyzed hydrogenation of pyrrolo[1,2-α]pyrazinium salts **24** provided a direct access to chiral derivatives of 1,2,3,4-tetrahydropyrrolo[1,2-α]pyrazine **26** with enantioselectivity up to 95% [26] (Scheme 8). A key feature of the reaction is the addition of cesium carbonate, which increases conversion and prevents racemization of the products (17 substrates were used in the work).

**Scheme 9:**
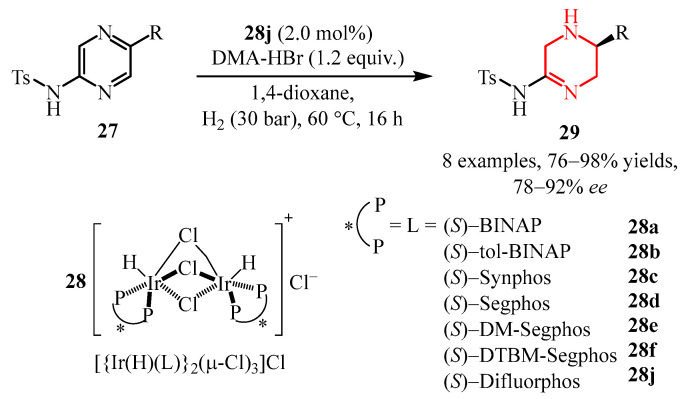
Ir-catalyzed asymmetric hydrogenation of tosylamido-phenyl pyrazine, according to [29]. R = *p*-MeOC_6_H_4_, *p*-CF_3_C_6_H_4_, *p*-CO_2_MeC_6_H_4_, *m*-MeOC_6_H_4_, *m*-CF_3_C_6_H_4_, *o*-MeC_6_H_4_, cyclopentyl, *n*-hexyl; *—optically active center.

A method for the asymmetric catalytic hydrogenation of pyrazines **27** in the presence of iridium complexes **28**, dioxane, and Brønsted acid composition was reported in [29] (Scheme 9). The reaction proceeded with acceptable selectivity to deliver the products **29** in moderate to good yields. It should be noted that the authors optimized the conditions of the studied process in the presence of seven Ir complexes (**28**) (Scheme 9) and thirteen additive compositions, namely, lutidine·HCl, lutidine·HBr, lutidine·HI, lutidine·C_6_F_5_COOH, lutidine·TsOH, 2,6-(*t*-Bu)_2_pyridine·HBr, aniline·HBr, *p*-MeO-aniline·HBr, *p*-CF_3_-aniline·HBr, *N*-methylaniline·HBr, *N*,*N*-dimethylaniline·HBr, *N*,*N*-dimethylaniline·HCl, and *N*,*N*-dimethylaniline·HI. The combination of **28j** and DMA·HBr was found to be optimal.

**Scheme 10:**
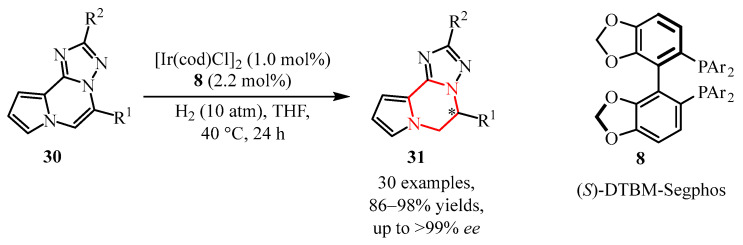
Ir-catalyzed asymmetric hydrogenation of 2,5-diaryl pyrrolo [1,2-*a*][1,2,4]triazolo [5,1-*c*]pyrazines, according to [45]. R^1^ = Ph, OMe, MeO-C_6_H_4_, and others; R^2^ = Ph, CH_3_-C_6_H_4_, 3-CH_3_O-C_6_H_4_, 4-CH_3_O-C_6_H_4_, 3-F-C_6_H_4_, 4-Cl-C_6_H_4_, 4-OH-C_6_H_4_, *i*-Pr-Ph, and others; *—optically active center.

The possibility of pyrazine ring saturation was also shown in the study by the authors of [45], which was devoted to the efficient synthesis of chiral polyheterocyclic compounds with several nitrogen atoms in the presence of an iridium complex. It is noteworthy that without additives, a number of chiral 2,5-disubstituted 5,6-dihydropyrrolo[1,2-α][1,2,4]triazolo[5,1-*c*]pyrazines **31** were obtained in high yields (86–98%), and excellent enantioselectivity was achieved (up to >99% *ee*) (Scheme 10).

The asymmetric hydrogenation of pyrazine derivatives in the presence of iridium catalysts was described by Stoltz et al. [23]. Also, for this purpose, ruthenium [46] and palladium [47,48] catalysts were employed. All the above versions of hydrogenation were implemented in the presence of molecular hydrogen, and under pressure of 20–80 bar, depending on the catalyst and the nature of the substrate. In addition, the reaction required a long time, which can be considered a significant disadvantage of the process.

### 2.2. Other Methodology of the Saturation

#### 2.2.1. Pyrimidine Moiety

**Scheme 11:**
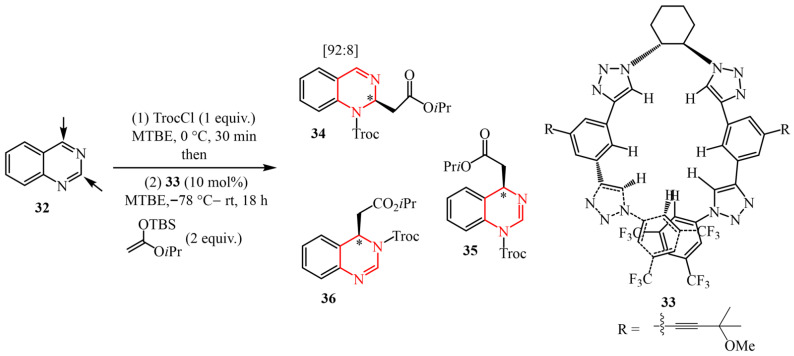
Enantioselective nucleophilic dearomatization of quinazoline using an organocatalyst, according to [31]; *—optically active center.

The first anion-binding organocatalyzed enantioselective Reissert-type dearomatization of pyrimidines **32** was documented in [31] (Scheme 11). Tetrakistriazole-based H-bond donor catalysts (TrocCl, CbzCl, and MeOCOCl) were more effective than other known hydrogen-bond donors, providing the corresponding products in high regioselectivities and up to 96 (**34**):4 (**35**) er. The formation of possible isomer **36** was not observed.

Unfortunately, the reaction requires a rather complex multicomponent catalytic system, which can be considered a significant disadvantage. In addition, during the second stage of implementation of the methodology, the temperature must be lowered to −78 °C.

**Scheme 12:**
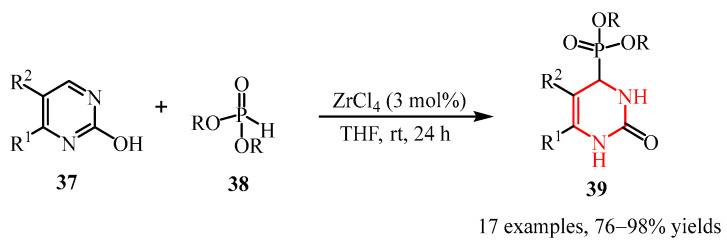
ZrCl_4_-catalyzed nucleophilic dearomatization of 2-hydroxy-pyrimidines with phosphites, according to [37]. R^1^ = Ph, 3-Me-C_6_H_4_, 4-Me-C_6_H_4_, 3-Cl-C_6_H_4_, 4-Cl-C_6_H_4_, 3-MeO-C_6_H_4_, 2,4-F_2_C_6_H_3_, Me; R^2^ = Me, Ph, 3-Me-C_6_H_4_, 4-Me-C_6_H_4_, 3-Cl-C_6_H_4_, 4-Cl-C_6_H_4_; and R = Me, Et, *i*-Pr.

A method for the catalytic nucleophilic dearomatization of 2-hydroxypyrimidines **37** using ZrCl_4_ as a catalyst and dialkyl phosphite **38** as a nucleophile was developed [37]. The reaction was carried out under mild conditions (Scheme 12).

The method presented in [37] is simple and does not require the use of expensive reagents other than zirconium salt. It can be performed at room temperature, but requires a longer reaction time.

#### 2.2.2. Pyrazine Moiety

Crabtree et al. [48] developed an interesting method for the regioselective reduction of *N*-heteroaromatic compounds. The method comprises the combination of hydrosilylation using 3 mol% [Rh(cod)(PPh_3_)_2_]PF_6_ and 4 eq. silane in toluene at room temperature, with subsequent hydrogenation with hydrogen transfer using 1 mol% [Ir(cod)(NHC)PPh_3_]BF_4_ in isopropanol with 0.5 eq. K_2_CO_3_ at 82 °C. According to the authors of [48], 1.00 mmol of pyrazine was fully hydrogenated within 24 h. Unfortunately, the authors did not provide data on the possibility of obtaining optically pure products using this method.

**Scheme 13:**
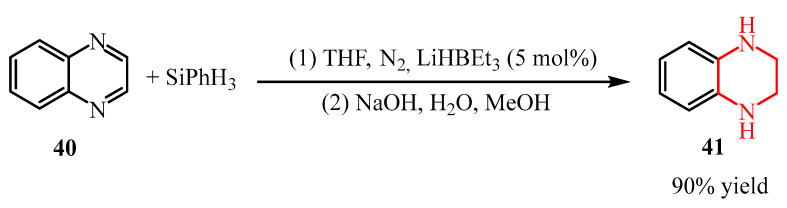
LiHBEt_3_-catalyzed transition and metal-free hydrosilylation of a quinoxaline with 1,2,3,4-tetrahydroquinoxaline, according to [49].

The authors of [49] demonstrated that the dearomatization of *N*-heteroarenes, including those containing a pyrazine fragment **40**, can be achieved using a LiHBEt_3_–SiPhH_3_-derived composition; the yield of the target products **41** reached 90% (Scheme 13). A plausible reaction mechanism was described based on DFT calculations (without providing a scheme).

**Scheme 14:**
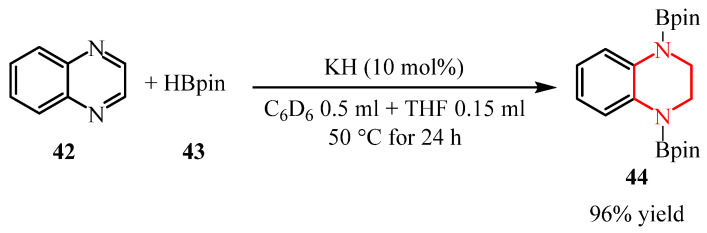
Catalytic 1,2-regioselective dearomatization of quinoxaline via a hydroboration, according to [33].

Liu et al. [50] accomplished the regioselective hydroboration of the pyrazine fragment **42** with pinacolborane **43** (HBpin) in the presence of commercially available potassium-based catalysts to furnish 1,4-regioselective hydroborated products **44** in quantitative yields (Scheme 14). The pyrimidine ring can also undergo hydroboration in this manner to give the target product in 96% yield.

The saturation of the pyrazine ring occurs in a similar way in the presence of such systems as I_2_-HBpin [51] and PhSiH_3_-HBpin [52]. In addition, it was shown that the PhSiH_3_-HBpin composition was efficient in the saturation of the pyrimidine.

A general approach to the dearomatizing hydroboration of *N*-heterocycles (including those with a pyrazine ring) using 31 high-performance catalytic systems based on d- and f-element complexes was outlined in a remarkable paper [53]. The optimal conditions for each catalytic composition, as well as the possible conversion mechanisms, were discussed in detail. It was emphasized that significant progress has been made in recent years within the domain of research concerning the hydroboration of *N*-heteroaromatic compounds using rare-earth catalysts.

An efficient method for electrocatalytic hydrogenation of pyrazine to piperazine was disclosed in [54]. The reaction occurred at ambient temperature and pressure in the presence of an anion-exchange membrane electrolyzer equipped with a Rh/C cathode that also acted as a catalyst.

**Scheme 15:**
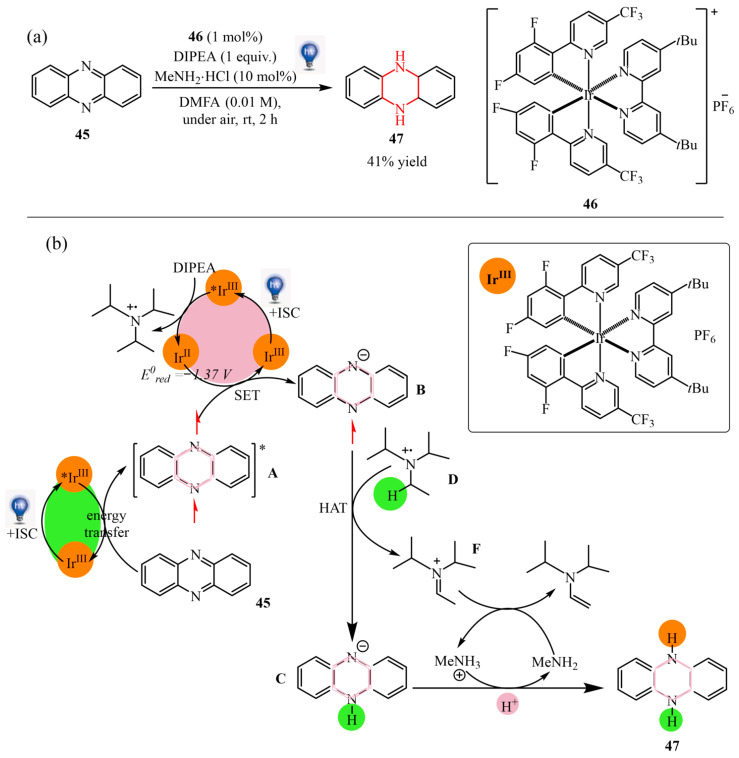
(**a**) Birch-type photoreduction of phenazine; (**b**) mechanistic proposal, according to [55]; *—optically active center.

A method for the direct reduction of the pyrazine ring **45** was developed [55]. It is noteworthy that this method is based on the Birch-type photoreduction catalysis under blue light irradiation, when diisopropylethylamine (1 eq.) was employed as the sacrificial electron donor, and 1 mol% Ir[dF(CF_3_)ppy]_2_-(dtbpy)PF_6_ **46** (where dF(CF_3_)ppy = 2-(2,4-difluorophenyl)-5-(trifluoromethyl)pyridine and dtbpy = 4,4′-di-*tert*-butyl-2,2′-bipyridine) was used as a photocatalyst, with DMFA (1 mL) used as a solvent. The reduction process was carried out at room temperature in air for 2 h (Scheme 15a). A general tentative mechanistic scheme was given (Scheme 15b). It was noted that pyridazine rings can also be reduced by this method, but the yield was half that obtained by reducing the corresponding pyrazine analogs.

The authors of [55] suggested that, when photoexcited by visible light **46**, the lowest triplet excited state, *IrIII, sensitizes the formation of the triplet state of the heteroarene* **A**. Consequently, one blue photon (455 nm of 2.72 eV) is used for the energy transfer process (Dexter energy transfer). In parallel, a second blue photon, excited by *IrIII, is quenched by DIPEA to form the IrII complex and the radical cation of DIPEA **D**. The excited heteroarene* **A** is reduced by the IrII complex to form heteroarene^•−^ **B**, which extracts a hydrogen atom from the radical cation of DIPEA **D**, producing the heteroarene^–^ carbanion **C**. Finally, protonation from MeNH_3_Cl or the iminium ion of DIPEA yields the reduced product **47**. The evidence obtained by the authors of [55] using UV–visible and EPR spectroscopy methods partly confirms their conclusions.

#### 2.2.3. Triazine Moiety

**Scheme 16:**
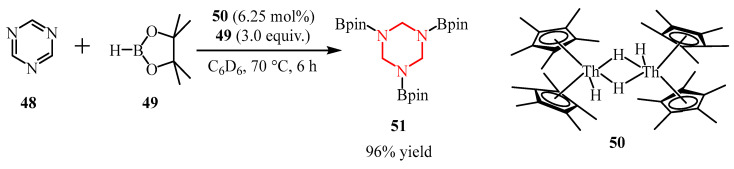
Catalytic 1,2-regioselective dearomatization of *N*-heteroaromatics via hydroboration, according to [33].

The method for the saturation of the 1,3,5-triazine **48** ring through the hydroboration reaction under mild conditions in the presence of thorium complex **50** (Scheme 16) was described in [33].

## 3. Construction of the Six-Membered Nonaromatic Polyazole Cores

This section discusses approaches to the construction of polyazocyclic compounds with fully or partially saturated *N*-containing fragments, that is, containing one double bond or containing no multiple bonds at all. The binding of several molecules that results in the formation of six-membered polyazocyclic compounds containing two double bonds, with (as a rule) still-retained aromaticity, was surveyed in the first part of this review.

### 3.1. Diazine Compounds

#### 3.1.1. Pyridazine Moiety

**Scheme 17:**
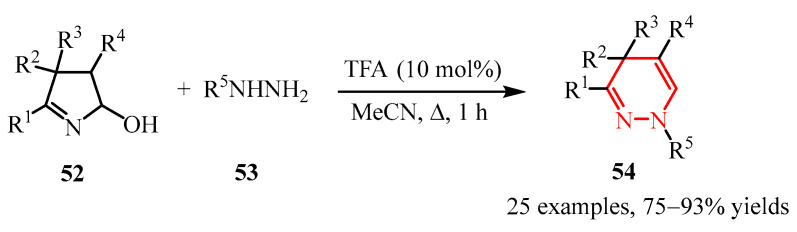
Metal-free selective synthesis of pyridazines, according to [56]. R^1^ = aryl, heteroaryl; R^2^ − R^3^ = alkyl, cycloalkyl; and R^4^ = H, Ph; R^5^ = alkyl, aryl, heteroaryl, Ts.

The recyclization method can be used to obtain pyridazines by either contracting, reducing the ring (diazepinone [56]), or expanding (dimethylfuran [57]). Scheme 17 shows the recyclization of 5-hydroxy-Δ1-pyrrolines **52** with hydrazines **53** to give pyridazines **54**.

#### 3.1.2. Pyrimidine Moiety

**Scheme 18:**
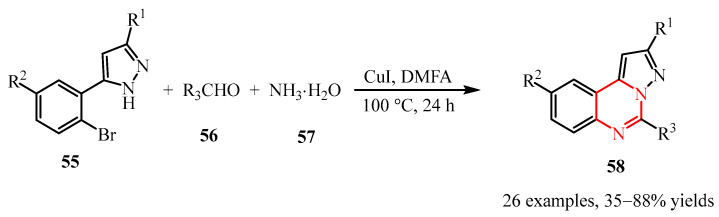
Cu-catalyzed tandem reaction of 5-(2-bromoaryl)-1*H*-pyrazoles with carbonyl compounds and aqueous ammonia, according to [58]. R^1^ = Me, Ph, 4-Cl-C_6_H_4_, 4-Me-C_6_H_4_; R^2^ = H, Cl, OMe; and R^3^ = alkyl, aryl, heteroaryl.

Guo’s group developed a Cu-catalytic approach to the preparation of pyrazolo[1,5-*c*]quinazolines **58** from substituted pyrazoles **55** (Scheme 18) [58].

During the chemical transformation shown in Scheme 18, in the resulting products 58, one of the nitrogen atoms of the nonaromatic six-membered ring also belongs to the adjacent five-membered aromatic cycle. As this does not formally contradict the main purpose of this review, the scheme was included.

**Scheme 19:**
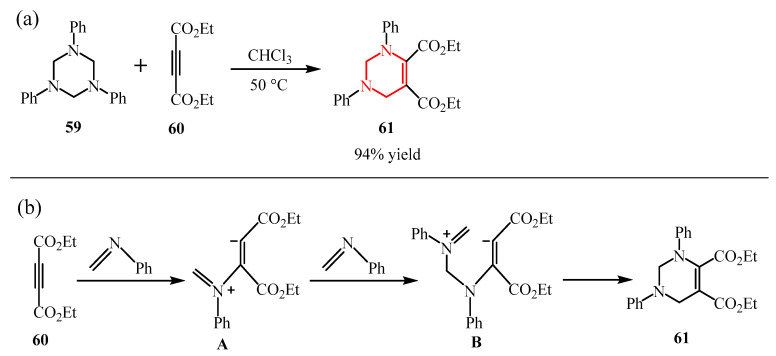
(**a**) [2+2+2] Cycloaddition reaction of 1,3,5-triphenyl-1,3,5-triazinane with but-2-ynedioate; (**b**) plausible mechanism, according to [59].

The [2+2+2]-cycloaddition reaction of 1,3,5-triazinane **59** with diethyl ester of acetylenedicarboxylic acid **60** was studied in [59] (Scheme 19a). The process proceeded under mild conditions, without a catalyst to deliver the products **61** in moderate to excellent yields (up to 99%). A tentative reaction mechanism was rationalized (Scheme 19b).

Based on the data of high-resolution mass spectrometry (HRMS), the authors of [59] suggested that the nucleophilic addition of in situ-obtained formalin to electron-deficient alkynes **60** generates Huisgen’s 1,4-dipoles **A** ([M]^+^ = 276.1230), which interact with another formalin, forming an intermediate compound **B** ([M]^+^ = 381.1809). Then, intramolecular ring closure leads to the target product **61**.

**Scheme 20:**
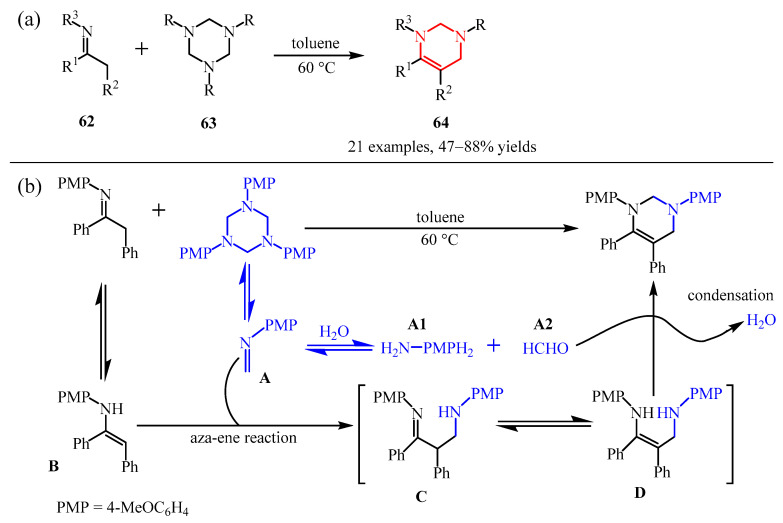
(**a**) Catalyst-free synthesis of tetrahydropyrimidines via formal [3+3]-cycloaddition of imines with 1,3,5-hexahydro-1,3,5-triazines; (**b**) plausible mechanism, according to [60]. R = 4-MeO-C_6_H_4_; R^1^ = Ph, 4-Me-C_6_H_4_, 4-Br-C_6_H_4_; R^2^ = Me, Bu, Ph, 4-BrC_6_H_4_, 2,4-Cl_2_C_6_H_4_; and R^3^ = 4-MeO-C_6_H_4_.

Polysubstituted tetrahydropyrimidines **64** can be synthesized by the protocol published in [60]. The facile, straightforward, and environmentally benign reaction proceeds via a formal catalyst-free [3+3]-cycloaddition of imines **62** to 1,3,5-triazines **63** (Scheme 20a). The highest yields were achieved in toluene at 60 °C. A plausible reaction mechanism was presented (Scheme 20b). In addition, it was shown that the process can be scaled up on a gram level.

The authors of [60] suggested that dissolution of 1,3,5-triazine **63** in a solvent would result in the formation of formalimine **A**, which could further decompose into 4-methoxyaniline **A1** and formaldehyde **A2** due to the presence of a small amount of water in the solvent. Imine **62** could isomerize into enamine **B** in the reaction system. The whole process could be caused by a formal azaene-type reaction between enamine **B** and formalimine **A**, affording active intermediates β-aminoimine **C** or 1,3-diamine **D**. These intermediates then react with the in situ-formed formaldehyde to form the condensation product of tetrahydropyrimidine **64** and release one molecule of water into the reaction system. Unfortunately, no detailed description of the role of water was presented in [60], nor was there any evidence of formaldehyde formation.

**Scheme 21:**
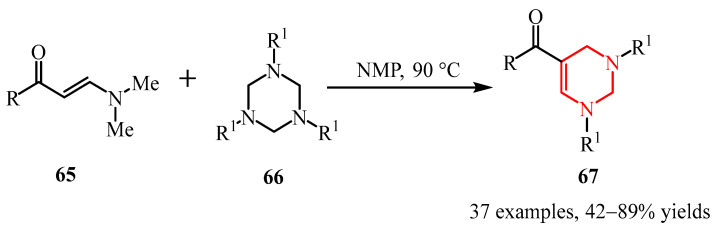
[4+2] Cycloaddition of enaminones with 1,3,5-triazinanes, according to [61]. R = alkyl, aryl; R^1^ = Bn, Ph.

The 1,2,3,4-tetrahydropyrimidines **67** bearing a partially saturated aromatic ring were also prepared in [61] (Scheme 21). The substrate scope of this reaction was evaluated using more than 37 substrates. Some of the products showed potential antitumor activity. It was demonstrated that the formation of the tetrahydropyrimidines **67** is a temperature-controlled process that requires no special additives. The utilization of *N*-methylpyrrolidone in the reaction is a notable benefit of this protocol.

**Scheme 22:**
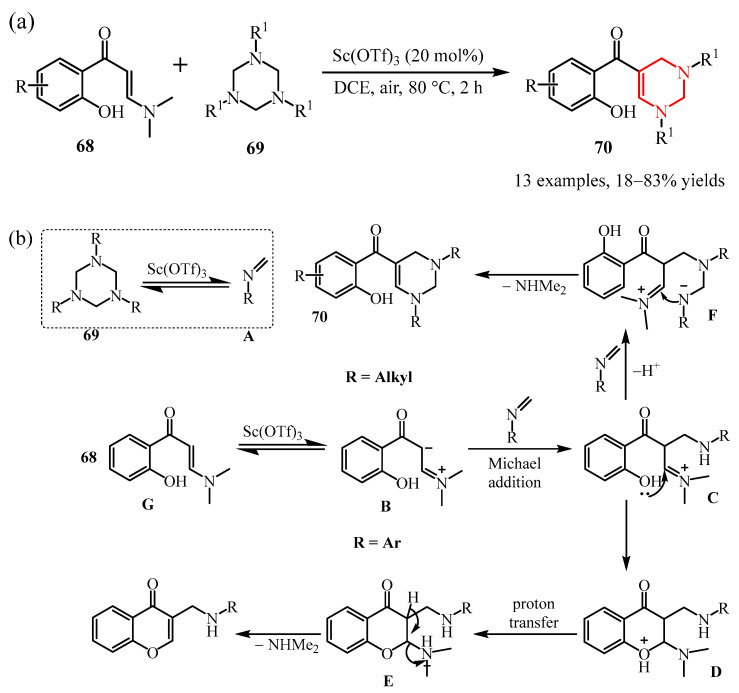
(**a**) Sc-Catalyzed tandem cyclization of *o*-hydroxyphenyl enaminones with 1,3,5-triazinanes; (**b**) proposed mechanism, according to [62]. R = F, Cl, Br, Me, MeO; R^1^ = Bn, Ph, and others.

The tetrahydropyrimidines **70** were obtained in moderate to good yields using the Sc(OTf)_3_ complex as a catalyst (Scheme 22a) under relatively mild conditions. A possible reaction mechanism was provided in [62] (Scheme 22b).

According to Scheme 22b, 1,3,5-triazinane **69** decomposes under the action of the Lewis acid Sc(OTf)_3_ to form *N*-phenylmethanimine **A**, while enaminone **G** is transformed to its isomeric form **B**. Michael addition of the latter to imine **A** forms iminium **C**, and a subsequent intramolecular *O*-Mannich reaction generates oxonium ion **D** if R is an aryl group. Oxonium **D** undergoes proton transfer to intermediate **E** and then, after elimination of dimethylamine, delivers the desired product **70**. Alternatively, the NH group in intermediate **C** can preferentially attack the alkylimine released from 1,3,5-trialkyl-1,3,5-triazinane to form intermediate **F**, which prevents the hydroxyl group from attacking its C=N bond to give the chromone skeleton. Finally, the nitrogen ion in intermediate **F** attacks its C=N bond to complete the intramolecular ring closure, producing the desired *N*,*N*-dialkyltetrahydropyrimidine **68** and releasing dimethylamine. Unfortunately, the authors of [62] did not provide any physicochemical evidence of this mechanism.

**Scheme 23:**
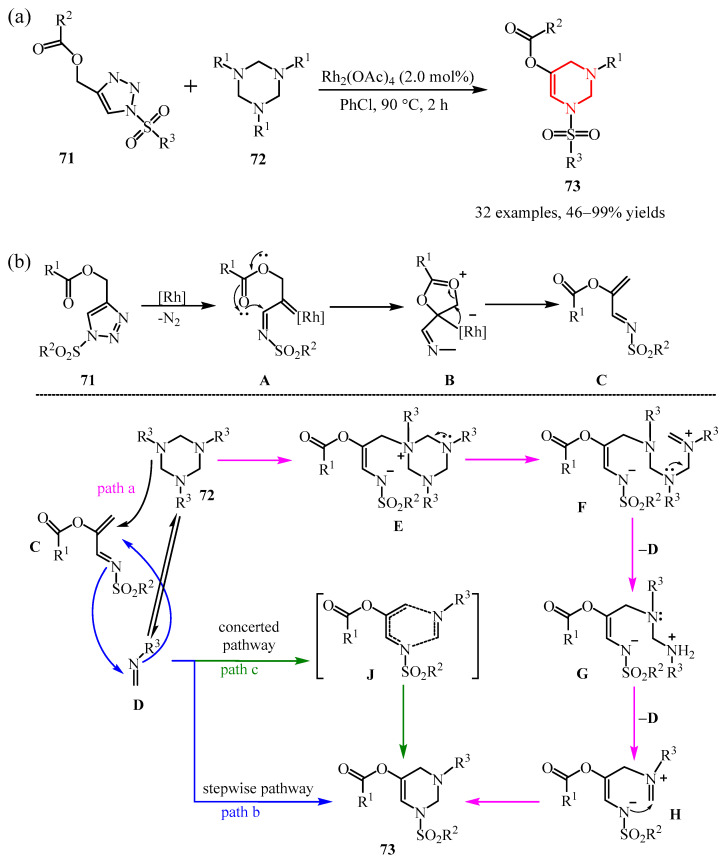
(**a**) Rh-catalyzed [4+2] annulation of ester-tethered triazole and hexahydro-1,3,5-triazines; (**b**) proposed mechanism, according to [63]. R^1^ = Me-C_6_H_4_, Ph, CO_2_Me-C_6_H_4_, MeO-C_6_H_4_, X-C_6_H_4_ (X = Cl, Br, F), and others; R^2^ = Me, 3-Me-C_6_H_4_, 3-MeO-C_6_H_4_, 3-CF_3_-C_6_H_4_, and others; and R^3^ = 3-Me-C_6_H_4_, *i*-Pr, Me, and others.

Li et al. [63] synthesized tetrahydropyrimidine derivatives **73** in good to excellent yields via rhodium(II) complex-promoted [4+2]-annelation of 1-sulfonyl-1,2,3-triazoles **71** with hexahydro-1,3,5-triazines **72** under relatively mild conditions (Scheme 23a). A tentative reaction mechanism was described (Scheme 23b).

According to Scheme 23b, α-iminorhodium carbene **A**, which can be readily prepared from 1-sulfonyl-1,2,3-triazole **71** in the presence of a rhodium(II) catalyst, can be converted to the α,β-unsaturated imine **C** via a 1,2-ether migration process. In route a, the nitrogen of hexahydro-1,3,5-triazine **72** attacks intermediate **C**. Subsequent rapid cleavage of the C–N bond and the release of formadimin **D** leads to intermediate **H**, and then 1,2,3,4-tetrahydropyrimidine **73** can be obtained via the intramolecular cyclization. Meanwhile, at a high temperature, an equilibrium between hexahydro-1,3,5-triazine **72** and formadimin **D** is observed. Route b involves a stepwise [4+2]-annulation between **C** and formadimin **D**, starting from an aza-Michael reaction. In route c, a concerted mechanism can also explain the formation of the final product **73**. Based on the electronic influence of the aromatic ring on triazines and the result of the reaction of triazole with imine, the authors of [63] considered route c as a possible exception. And in this case, the proposed scheme of the mechanism was not confirmed by any evidence obtained through physical and chemical research.

**Scheme 24:**
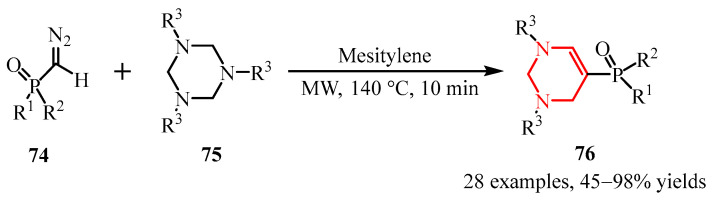
Annulations of phosphoryl diazomethanes and 1,3,5-triazinanes, according to [64]. R^1^, R^2^ = Ar, Alkoxy; R^3^ = aryl, alkyl.

The assemblies of 1,2,3,4-tetrahydropyrimidines assisted by microwave radiation or ultrasound were documented. For example, it was reported [64] that 5-phosphoryl-1,2,3,4-tetrahydropyrimidines **76** were synthesized under microwave irradiation conditions (Scheme 24). The reaction represents a sequence of 1,3,5-triazinane **75** fragmentation and tandem nucleophilic addition of the obtained formaldimines and phosphoryl diazomethanes **74**, followed by *N*,*N*-acetalization.

**Scheme 25:**
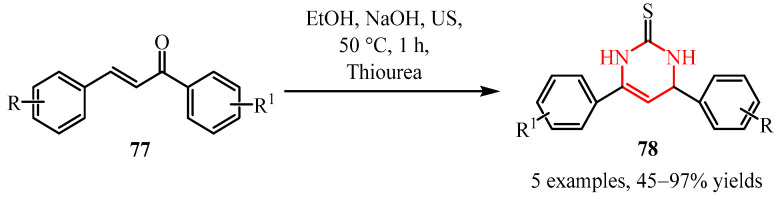
Synthesis of dihydropyrimidine-2(1*H*)-thiones, according to [65]. R = H, 4-Me, 4-Cl, 4-F; R^1^ = H, 2,4-OMe.

Furthermore, Grabowiecka et al. [65] developed an ultrasound-promoted method for the synthesis of dihydropyrimidines **78** (Scheme 25) with good to excellent yields.

**Scheme 26:**
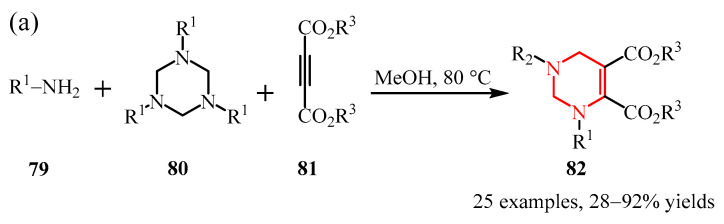

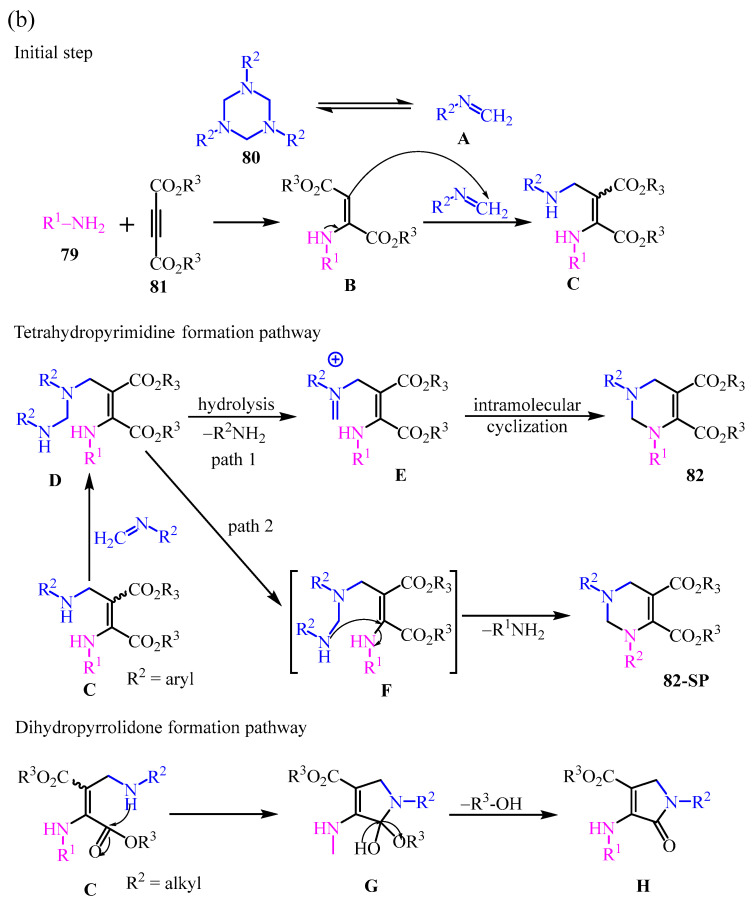
(**a**) Three-component reaction of amines, 1,3,5-triazinanes, and alkyne esters for the construction of tetrahydropyrimidine derivatives; (**b**) proposed mechanism, according to [66]. R^1^ = Bn, cyclophentyl, cyclopropyl, and others; R^2^ = Ph; R^3^ = Me.

Tetrahydropyrimidines **82** were prepared [66] under mild conditions via [2+1+3]-cycloaddition using aryl-substituted 1,3,5-triazinanes **80** (Scheme 26a) as three-atom synthons. A possible reaction mechanism was proposed (Scheme 26b).

According to the data obtained in [66], first, 1,3,5-triazinane **80** decomposes to the corresponding formaldimine **A**. Enamines **B** are obtained by hydroamination of amines **79** with alkyne ethers **81**. Then **B** undergoes nucleophilic attack by formaldimines **A** to form the intermediate product **C**. In the synthesis of tetrahydropyrimidines (tetrahydropyrimidine formation pathway), **C** attacks another formaldimine **A** to give the intermediate product **D**. After hydrolysis, intramolecular cyclization of **E** affords product **82** (path 1). If R^1^ is a nitro-substituted group, **82-SP** is produced by nucleophilic attack of a secondary amine and the eliminated nitroaniline (path 2). In the synthesis of dihydropyrrolidones (dihydropyrrolidone formation pathway), the nucleophilicity of the secondary amine **C** is enhanced by the attached alkyl group, which facilitates intramolecular addition to form **G**. The final product **H** is obtained by elimination of the alcohol.

**Scheme 27:**
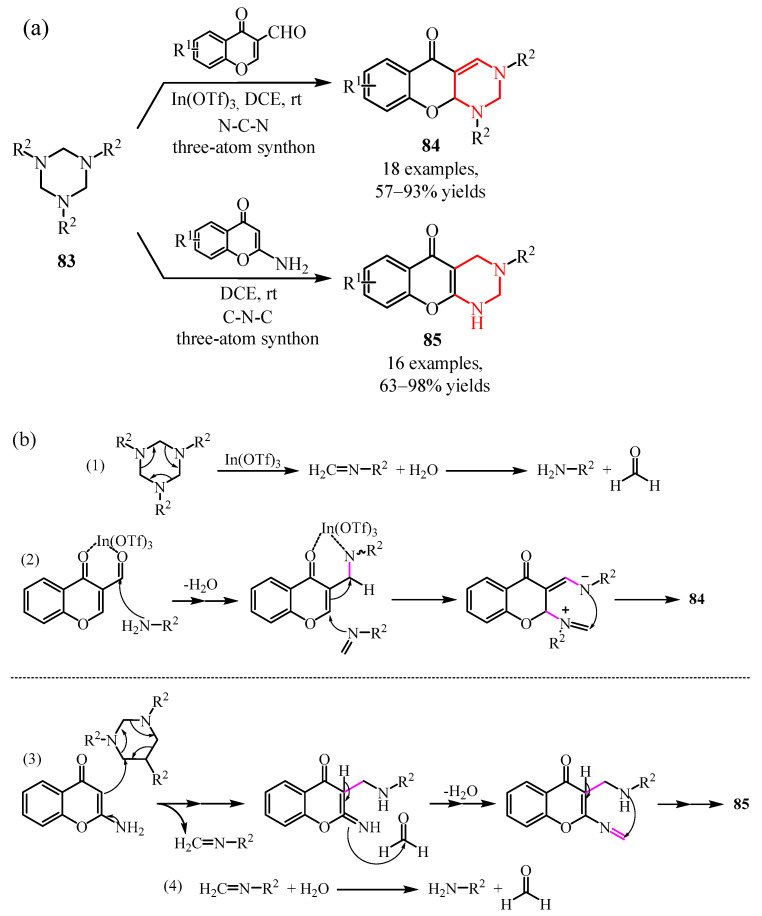
(**a**) Synthesis of chromeno [2,3-*d*]pyrimidin-5-one derivatives from 1,3,5-triazinanes; (**b**) proposed mechanism, according to [67]. R^1^ = H, Me, Cl, Br, F, and others; R^2^ = Me-C_6_H_4_.

Two synthetic protocols were reported for the preparation of chromeno[2,3-*d*]pyrimidin-5-ones using 1,3,5-triazinane as an N-containing building block via different reaction modes [67]. In the first case, the target product containing a partially saturated pyrimidine moiety **84** was obtained in the presence of an In(OTf)_3_ catalyst. The second protocol afforded the target product bearing an entirely saturated pyrimidine moiety **85** in 63–98% yield (Scheme 27a). A possible reaction mechanism was presented (Scheme 27b).

According to the data of [67], 1,3,5-triazinane under catalytic conditions in the presence of In(OTf)_3_ can be easily decomposed into three units of formaldehyde, which then react with trace amounts of water in a solvent to form amine and formaldehyde (Scheme 27(b1)). Notably, water will be regenerated from the subsequent condensation reaction, with the release of formaldehyde and amine. To obtain **84**, In(OTf)_3_ catalyzes the formation of imine and subsequent Michael addition of formaldehyde to the α,β-unsaturated imine moiety, and then the pyrimidine ring is closed by an *N*-Mannich reaction (Scheme 27(b2)), while 2-amino-4*H*-chromen-4-one can directly attack 1,3,5-triazinane to give the aminomethylation product, and the remaining 1,3,5-triazinane is cleaved to formaldehyde (Scheme 27(b4)). The resulting formaldehyde can yield formaldehyde via hydrolysis for the subsequent condensation reaction. The resulting intermediate then undergoes imine–enamine tautomerism/imine formation, followed by an intramolecular *N*-Mannich reaction to give compound **85** (Scheme 27(b3)).

**Scheme 28:**
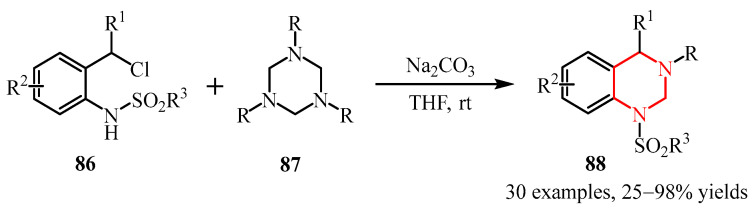
[4+2]-Cycloaddition of 1,3,5- triazinanes with aryl amides, according to [68]. R = Ph, Me-C_6_H_4_, X-C_6_H_4_ (X = F, Cl, Br), and others; R^1^ = H; R^2^ = H, Me; and R^3^ = Me-C_6_H_4_, F-Ph, NO_2_-Ph, and others.

The first example of an inverse-electron-demand [4+2]-cycloaddition of 1,3,5-triazinanes **87** (Scheme 28) was reported in [68]. The reaction occurred in mild conditions in the presence of bases such as Na_2_CO_3_, K_2_CO_3_, Cs_2_CO_3_, KOH, and NaOH, with Na_2_CO_3_ being the most efficient.

**Scheme 29:**
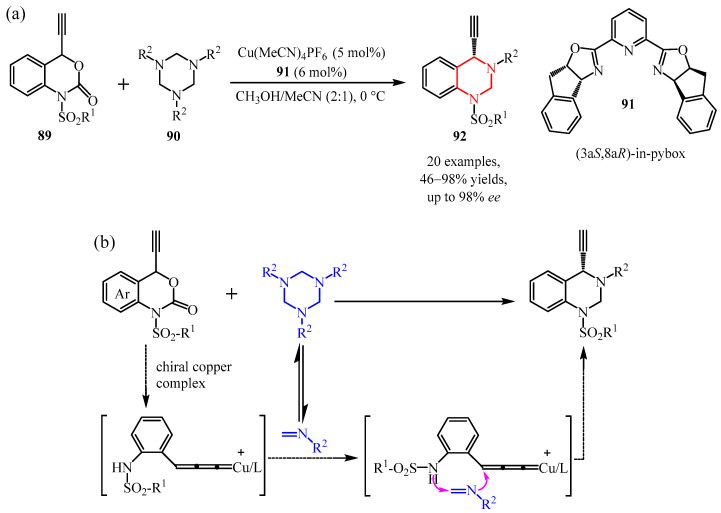
(**a**) Asymmetric [4+2]-cycloaddition of Cu−allenylidenes with hexahydro-1,3,5-triazines; (**b**) proposed mechanism, according to [69]. R^1^ = Me-C_6_H_4_, R^2^ = Bn, Me, Me-C_6_H_4_, X-C_6_H_4_ (X = Cl, Br, OMe), and others.

Sun et al. developed [69] a method for the asymmetric formal [4+2]-cycloaddition of copper–allenylidene to hexahydro-1,3,5-triazines **90** to produce chiral tetrahydroquinazolines **92** in moderate to good yields (up to 88%) and with high enantioselectivity (*ee* 98%) (Scheme 29a). A plausible reaction mechanism was proposed, a possible mechanism is described, and the prospects of the method developed were outlined (Scheme 29b).

Remarkably, in the asymmetric cycloaddition described by Scheme 29b, the reaction yields two amphiphilic dipolar intermediates generated in situ: copper complexes with allenidine and formyl imine. Copper with allenidine is captured by formaldimine, followed by cyclization, which gives a chiral product.

**Scheme 30:**
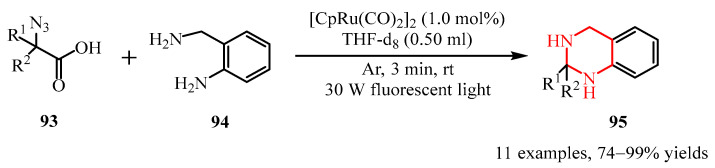
Synthesis of 1,2,3,4-tetrahydroquinazolines from α-azidocarboxylic acids and 2-(aminomethyl)aniline, according to [70]. R^1^ = H, Me, Ph; R^2^ = H, *n*-Bu, *i*Pr, C_6_H_4_-CH_2_-, *t*-Bu, and others.

The synthesis of 1,2,3,4-tetrahydroquinazolines **95** from α-azidocarboxylic acid **93** and 2-(aminomethyl)aniline **94** was accomplished in [70] (Scheme 30). The transformation was carried out in the presence of the ruthenium complex [CpRu(CO)_2_]_2_ under visible light and at room temperature to give the target products in almost quantitative yields.

**Scheme 31:**
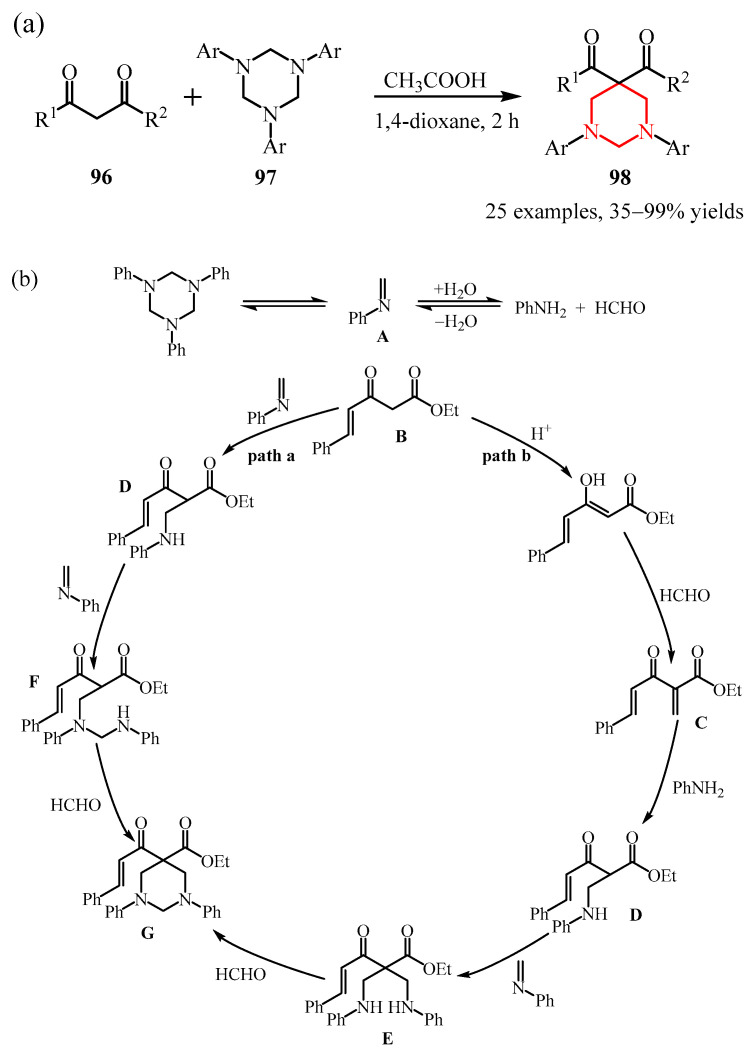
(**a**) Acetic acid promoted [2+2+1+1] cycloaddition of 1,3,5-triazinanes with active methylene; (**b**) proposed mechanism, according to [71]. R^1^ = Me, OEt, and others; R^2^ = Me, C_6_H_4_-C_2_H_2_, and others; and Ar = Ph, Me-C_6_H_4_, MeO-C_6_H_4_, X-C_6_H_4_ (X = Cl, F), and others.

A strategy for the preparation of hexahydropyrimidines (25 examples) via the cycloaddition of 1,3,5-triazinane to methylene-containing compounds was published in [71] (Scheme 31a). It was found that the reaction took place under mild conditions for 2 h to afford the products in up to a 99% yield. It was shown that this method can be scaled up to produce hexahydropyrimidines in gram quantities. A possible reaction mechanism was rationalized (Scheme 31b).

According to Scheme 31b, benzylimine **A** is formed from **97**, which is then hydrolyzed to give aniline and formaldehyde in the presence of traces of water. In path a, **B** sequentially reacts with benzylimine **A** to give intermediates **D** and **F**, respectively. In path b, C is formed by the condensation of **B** with formaldehyde. **C** then undergoes a Michael addition reaction with aniline to generate the key intermediate **D**, which is further trapped by benzylimine **A**, delivering **E**. Finally, product **G** is obtained by cycloaddition of **E/F** and in situ-generated formaldehyde. It is noteworthy that the possible key intermediates **C**, **D**, **E**, and **F** were detected by high-performance mass spectrometry: for **C** [M+H]^+^ = 231.1016, for **D** [M+H]^+^ = 324.1594, for **E** [M+H]^+^ = 429.2173, and for **F** [M+H]^+^ = 429.2173.

**Scheme 32:**
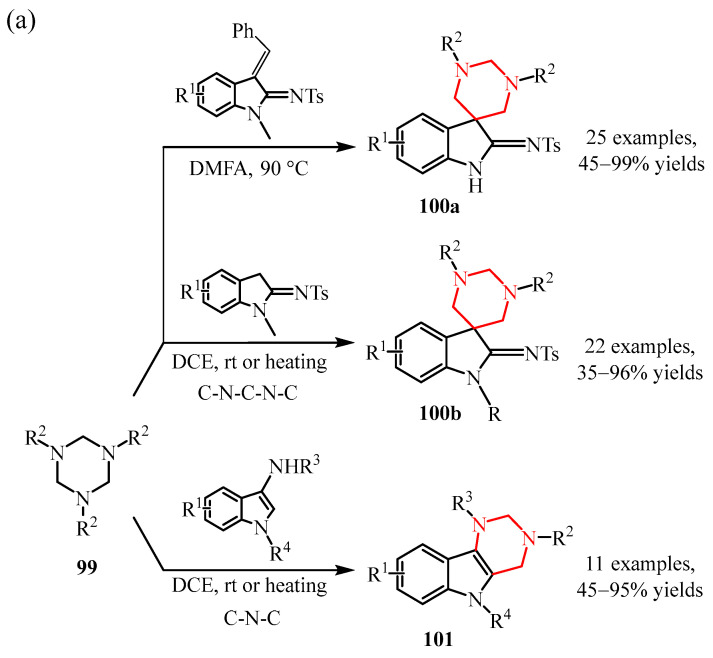

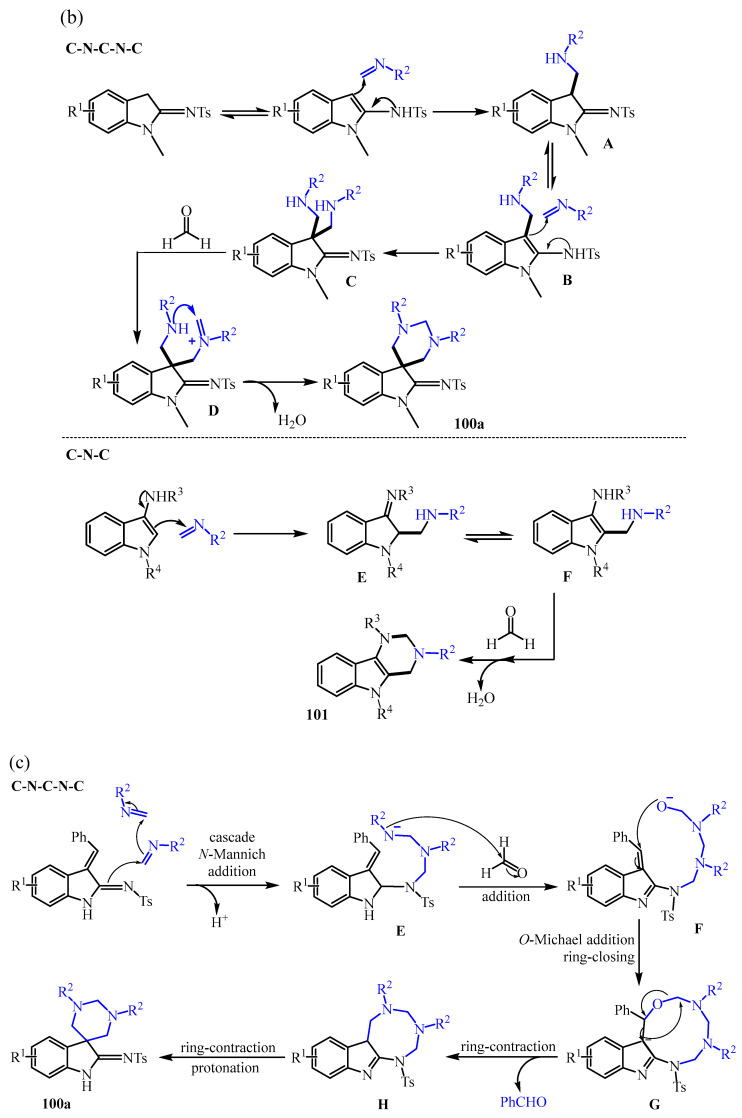
(**a**) Cyclization of indoline-derived azadienes with 1,3,5-triazinanes, according to [72,73]; (**b**) proposed mechanism, according to [72]; and (**c**) proposed mechanism, according to [73]. R = Me, H; R^1^ = H, Me, OMe, F, Cl, Br; R^2^ = Ph, Me-C_6_H_4_, and others; R^3^ = Ts, NO_2_-C_6_H_4_, and others; and R^4^ = Me, Ac.

A methodology for the preparation of hydropyrimidines by altering the position of the amino fragments was presented in [72,73]. The reactions were performed without catalysts and special additives. It was shown that two reaction directions are possible, in which either five atoms (i.e., C-N-C-N-C) or three atoms (i.e., C-N-C) of 1,3,5-triazine **99** can be sequentially introduced into annulation products (Scheme 32a). Possible mechanisms of these transformations were considered (Scheme 32b,c).

According to the data presented in [72], formaldimine could be obtained in situ from 1,3,5-triazinane, which is in equilibrium with formaldimine in the reaction system (Scheme 32b). Formaldehyde can be formed through the hydrolysis of formaldimine with traces of water, as reported in the works of previous authors. Then, 2-sulfonyliminoindoline reacts twice with the obtained formaldimin, introducing two aminomethyl groups at its 3-position, giving **C**. Subsequently, any of the obtained amino groups is condensed with formaldehyde, giving an iminium (e.g., **D**), which undergoes intramolecular *N*-Mannich annulation, to furnish pyrimidine-spiro-fused indolines **100b** (equation C-N-C-N-C). Condensed indoles and their analogs **101** are formed along the pathway when the activated amino group is in the 3-position of benzoheterocycles (equation C-N-C).

On the other hand, another mechanism exists for the formation of derivative **100** (**100a**) [73] (see Scheme 32c). According to this mechanism, indoline-derived azadienes can rapidly react with formaldimines to form intermediate **E**, which then transforms into intermediate F. A ring closure reaction then occurs to produce the ten-membered cyclic intermediate **G**. Further, a ring contraction reaction takes place through the elimination of one benzaldehyde molecule from intermediate **G** to give a new eight-membered cyclic intermediate **H**. The latter undergoes another ring contraction reaction to afford formally [1+5]-annulated indoline-spiro-hexahydropyrimidine products via the subsequent protonation.

**Scheme 33:**
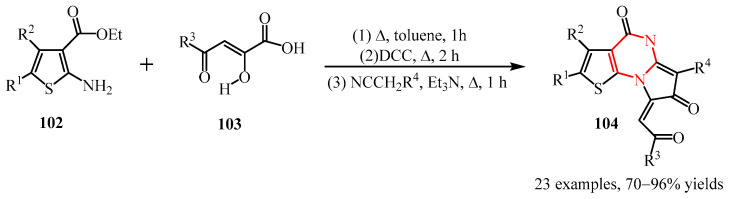
One-pot synthesis of thieno [3,2-*e*]pyrrolo [1,2-*a*]pyrimidine derivatives, according to [74]. R^1^ = alkyl, cycloalkyl; R^2^ alkyl, cycloalkyl, Ph; R^3^ = *t*-Bu, aryl, heteroaryl; and R^4^ = COOEt, CONH_2_, CN.

The cascade reaction of various Gewald aminothiophenes **102**, 2-hydroxy-4-oxobut-2-enic acid **103**, and cyanoacetic acid derivatives allowed the direct synthesis of various 1-(2-oxoethylidene)-2-oxothieno[3,2-*e*]pyrrolo[1,2-α]pyrimidines **104** (Scheme 33) [74].

**Scheme 34:**
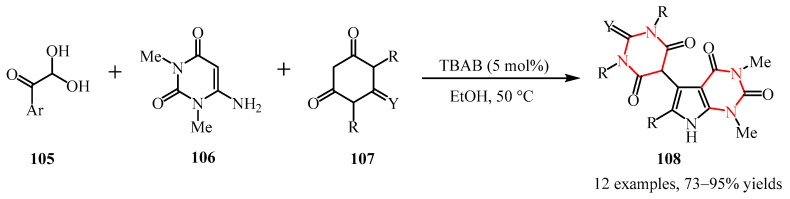
One-pot synthesis of pyrrolo [2,3-*d*]pyrimidine derivatives, according to [75]. Ar = Ph, 4-Me-C_6_H_4_, 4-Cl-C_6_H_4_, 4-NO_2_-C_6_H_4_; R = H, Me; Y = O, S.

Polyfunctionalized pyrrolo[2,3-*d*]pyrimidine derivatives **108** were synthesized in high yields [75] via a one-pot three-component reaction of arylglyoxal **105**, 6-amino-1,3-dimethyluracil **107**, and barbituric acid derivatives **106** (Scheme 34).

#### 3.1.3. Pyrazine Moiety

**Scheme 35:**
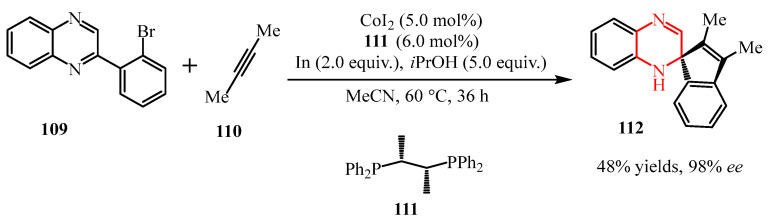
Enantioselective dearomative [3+2] annulation reaction of quinoxaline and 2-butyne, according to [76].

In 2022, Jia et al. [76] reported highly interesting dearomatized [3+2] umpolung-annulation of a variety of *o*-halogenphenyl-substituted *N*-heteroarenes **109**, including electron-deficient pyridines with alkynes **110** (Scheme 35). It was underlined that this strategy provided an efficient approach to a large number of chiral *N*-heterocyclic compounds **112** containing a spiro-quaternary stereocenter in moderate to good yields and with high enantioselectivity. The method tolerates both internal and terminal alkynes. A possible reaction mechanism was proposed.

**Scheme 36:**
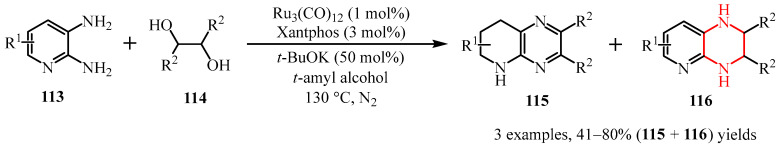
Ru-catalyzed annulative transfer hydrogenation strategy based on *N*-heteroaryl diamines and vicinal diols, according to [77]. R^1^ = H, Me, and others; R^2^ = Me, Ph, H, and others.

It was revealed that a ruthenium-catalyzed annulative transfer hydrogenation strategy provided direct access to tetrahydrocondensed pyrazine derivatives from *N*-heteroaryldiamines **113** and vicinal diols **114 [77]** (Scheme 36). The only by-product of this process was water.

**Scheme 37:**
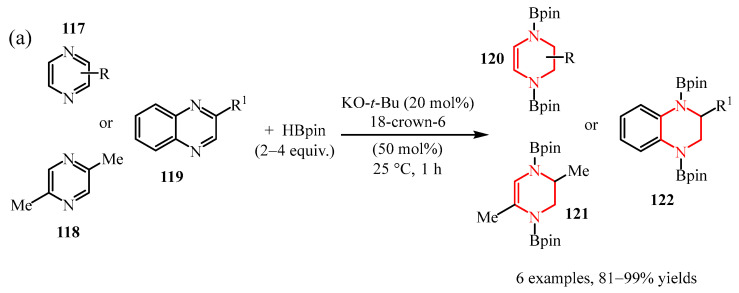

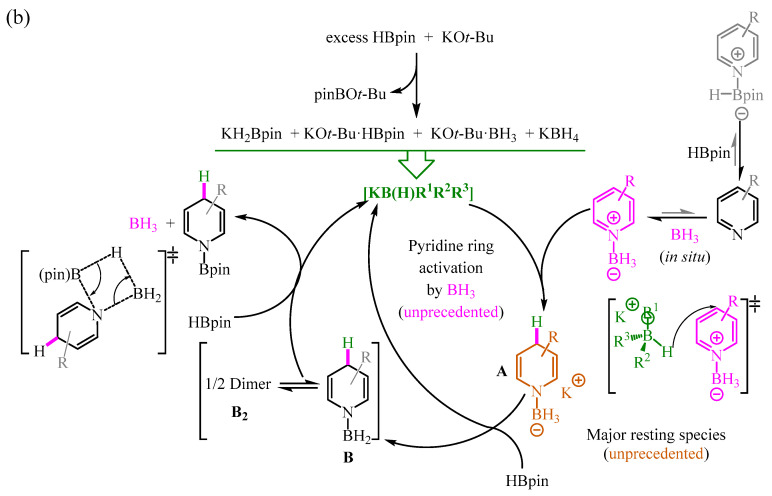
(**a**) Alkoxide-promoted selective hydroboration of *N*-heteroarenes; (**b**) proposed catalytic cycle, according to [78]. R = H, Me, Et.

Chang et al. [78] demonstrated the possibility of saturation of pyrazine (**117–119**) rings in the presence of alkoxide catalyst KO-*t*-Bu (Scheme 37a). A possible mechanism of this process was discussed (Scheme 37b).

Scheme 37b was proposed [78] based on experimental observations. It is suggested that 1,4-hydroboration of pyridines is realized via a KO*t*-Bu-promoted catalytic pathway. Initially, KO*t*-Bu reacts with HBpin (in excess) to rapidly establish a pre-equilibrium between several borohydride species, including KH_2_Bpin, KO*t*-Bu·HBpin, KO*t*-Bu·BH_3_, and KBH_4_. The in situ-formed pyridine-BH_3_ adduct undergoes nucleophilic hydride attack by the borohydride species to form 1,4-dihydropyridylborohydride (**A**), which is the major residual species. Hydride transfer from **A** to HBpin is proposed to occur slowly, reducing the borohydrides to release 1,4-dihydropyridylborane, presumably in the dimeric form (**B_2_**). Finally, monomeric **B** reacts with HBpin to give the product *N*-Bpin-1,4-dihydropyridine, releasing BH_3_, presumably via α-linkage metathesis.

**Scheme 38:**
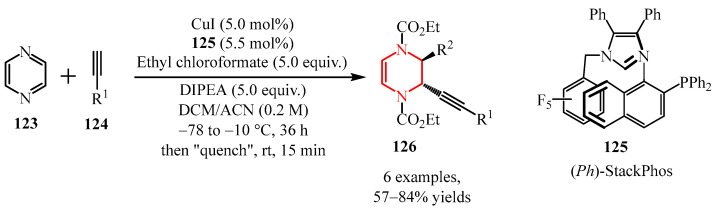
Enantioselective catalytic dearomatization of pyrazine, according to [35]. R^1^ = Ph, MeO-C_6_H_4_, and others; R^2^ = H, OH, Ac, and others. ‘Quench’ is a procedure that stops (or quenches) the reaction. In the standard version, this is achieved by adding 2.0 mL of methanol to the reaction solution using a syringe and stirring for 15 min.

In 2024, an enantioselective dearomatization of pyrazine **123** with terminal alkynes **124** in the presence of a copper complex (Scheme 38) using chloroformate as an activating reagent and (*R*)-StackPhos **125** as a chiral ligand was disclosed [35].

This method enables the synthesis of 2,3-disubstituted tetrahydropyrazines as a single diastereomer with high enantiomeric excess. These products can also be further converted into chiral C-substituted piperazines and C1-asymmetric 1,2-diamines. Mechanistic studies showed that the non-innocent role of the chloride ion can prevent the alkynylation of the second iminium, thus creating conditions for subsequent functionalization.

**Scheme 39:**
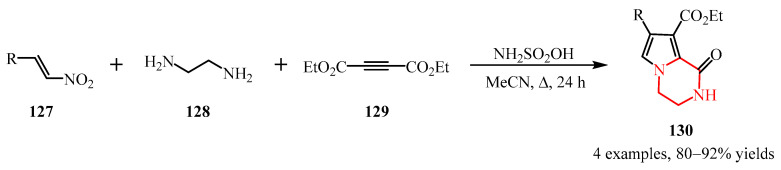
One-pot synthesis of pyrrolo[1,2-*a*]pyrazines, according to [79]. R = Ph, 4-MeC_6_H_4_, 4-ClC_6_H_4_, 4-NO_2_C_6_H_4_.

Moradi et al. demonstrated that sulfamic acid reacted efficiently with β-nitrostyrenes **127**, ethylenediamine **128**, and dimethylacetylenedicarboxylate **129** in a one-pot manner to generate pyrrolo[1,2-*a*]pyrazines **130** in good yields (Scheme 39) [79].

The main drawback of the methods described in this section is that they are multicomponent.

### 3.2. Triazine Compounds

#### 3.2.1. 1,2,3-Triazines

**Scheme 40:**
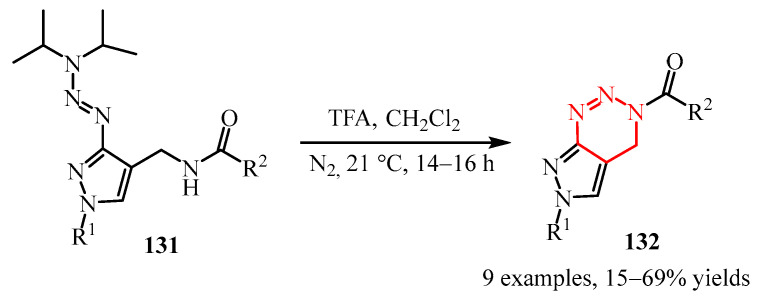
Synthesis of 3,4-dihydrobenzo[*d*][1,2,3]triazine derivatives, according to [80]. R^1^ = alkyl, cycloalkyl, Bn, 4-Me-Bn, 3,5-F-Bn; R^2^ = Me, Ph, *i*Bu.

H. Wippert et al. reported the five-step synthesis of compounds **132**. The final step involved the cleavage of the triazene amide link from **131**, followed by cyclization to pyrazolo[3,4-*d*][1,2,3]triazine compounds **132** (Scheme 40) [80].

**Scheme 41:**
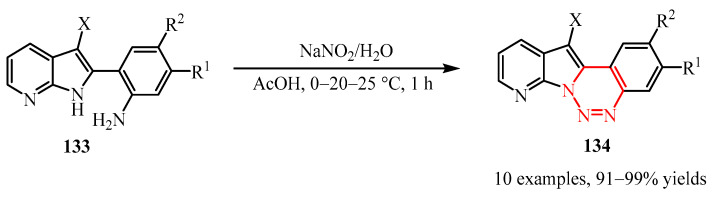
Synthesis of substituted 12-nitrosopyrido [3′,2′:4,5]pyrrolo [1,2-*c*][1,2,3] benzotriazines, according to [81]. R^1^ = H, Cl, Me, OMe; R^2^ = H, Cl, OMe; and X = Br, NO_2_.

Pyrrolo[1,2-*c*][1,2,3]triazines are a class of heterocycles that have received only limited attention from researchers. In 2014, two coauthors of this paper synthesized pyrido [3′,2′:4,5]pyrrolo[1,2-*c*][1,2,3]benzotriazines **134** by intramolecular cyclization of diazonium intermediates **133** (Scheme 41) [81]. The synthesis of pyrrolo[1,2-*c*][1,2,3]benzotriazines from 2-(1*H*-indol-2-yl)aniline was also disclosed [82].

In Scheme 41 (as in Scheme 18), one of the nitrogen atoms of the six-membered nonaromatic ring also belongs to the conjugated five-membered cycle. However, this fact does not formally contradict the main purpose of this review, which is why Scheme 41 was also included.

#### 3.2.2. 1,2,4-Triazines

**Scheme 42:**
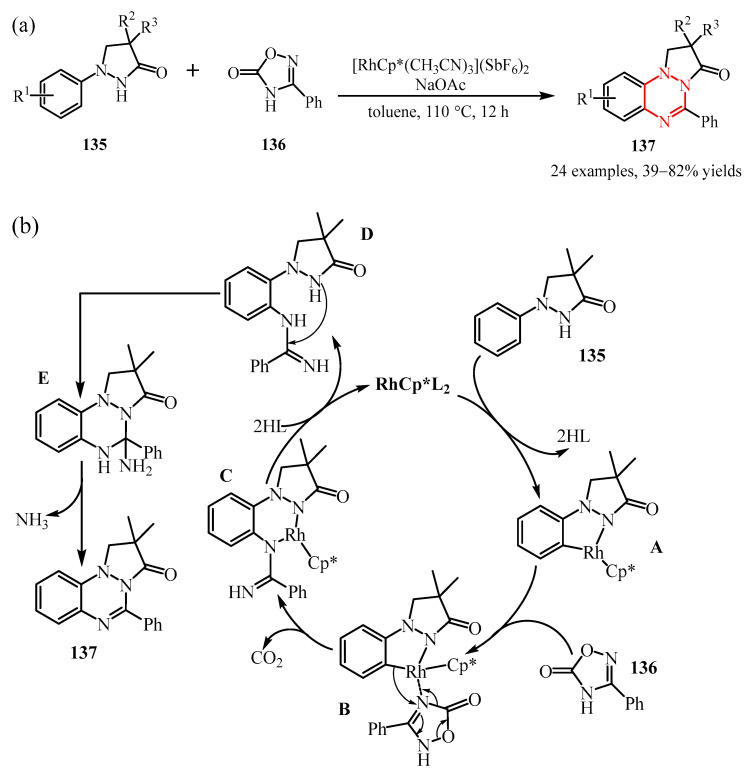
(**a**) C−H/N−H bond functionalization of 1-phenylpyrazolidinones with oxadiazolones; (**b**) plausible mechanism, according to [83]. R^1^ = H, Me, Et, OMe, OBn, F, Cl, Br, CN, CF_3_, and others; R^2^ = H, Me; and R^3^ = H, Me; *—optically active center.

The authors of [83] demonstrated for the first time that the C-H bond can be activated using the [RhCp*(CH_3_CN)_3_](SbF_6_)_2_ complex to produce pyrazolidinone-condensed benzotriazines **137** (Scheme 42a). It was noted that this methodology enabled the preparation of valuable products in high yields from readily available substrates under redox-neutral conditions. A plausible mechanism of the reaction was considered. In addition, the process was scaled up (Scheme 42b).

According to Scheme 42b, the first step involves metallation of the C−H/N−H bond of model compound **135** with Rh(III) to form a five-membered rhodacycle **A**. Then, coordination of **A** with **136** produces intermediate **B**, which then undergoes migratory insertion with simultaneous ring opening of the oxadiazolone moiety and displacement of CO_2_, forming a six-membered rhodacycle **C**. Subsequent protodemetallation of intermediate **C** leads to the formation of the aminated intermediate **D** and regeneration of the active catalyst. In the next step of this cascade reaction, *N*-nucleophilic attack of the pyrazolidinone fragment on the in situ-established amidine fragment gives the intermediate compound **E**. Finally, ammonia is eliminated from **E** to deliver product **137**. Unfortunately, as in many other works cited above, the authors of [83] proposed Scheme 42b based on the identification of the reaction products and analysis of literature data. No physicochemical studies of the process itself in situ were carried out.

**Scheme 43:**
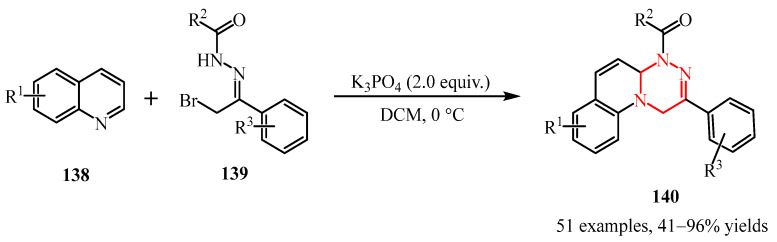
Dearomative [4+2] annulation of electron-poor *N*-heteroarenes with azoalkenes, according to [84]. R^1^ = H, Br, F, CN; R^2^ = H, Ph, OCH_3_, O-*t*-Bu, O-Bn; and R^3^ =Cl, H.

Guo et al. [84] reported on the dearomatization via [4+2] annulation of *N*-heteroarenes **138** with azoalkenes **139** obtained in situ from α-halohydrazones **140** under mild conditions (Scheme 43). The application scope of this reaction was evaluated using scaled-up synthesis and product derivatization.

**Scheme 44:**
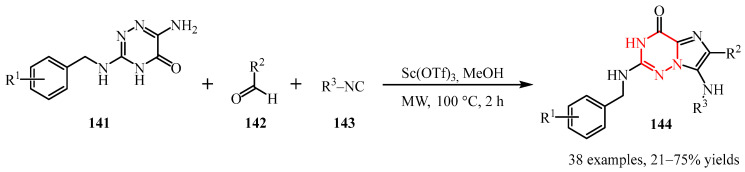
One-pot synthesis of novel 4-aza-9-deaza-guanine isosteres, according to [85]. R^1^ = H, 4-OMe, 2,4-(OMe)_2_, 3,4,5-(OMe)_3_; R^2^ = Ph, 4-Cl-C_6_H_5_, 4-MeO-C_6_H_5_, 2,4-(OMe)_2_-C_6_H_5_, 4-Br-C_6_H_5_, and others; and R^3^ = Ph, *i*-Pr, and others.

Dömling et al. [85] implemented microwave-assisted synthesis of the triazine derivatives **144** (Scheme 44).

**Scheme 45:**
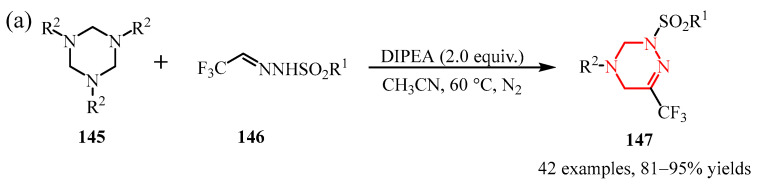

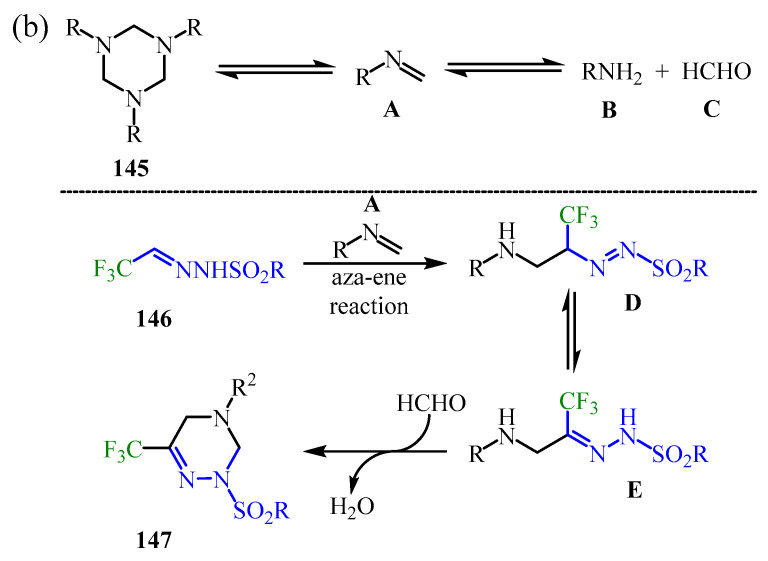
(**a**) Metal-free [2+1+3] cycloaddition of trifluoroacetaldehyde *N*-sulfonylhydrazones with hexahydro-1,3,5-triazines; (**b**) plausible mechanism, according to [86]. R^1^, R^2^ = Aryl, alkyl.

The synthesis of trifluoromethylated 2,3,4,5-tetrahydro-1,2,4-triazines **147** in 81–97% via [2+1+3]-cycloaddition of trifluoroacetaldehyde *N*-sulfonylhydrazones **146** to hexahydro-1,3,5-triazines **145** was carried out in [86] (Scheme 45a). The reaction tolerated a wide range of substrates, including aryl, benzyl, and alkyl hexahydro-1,3,5-triazines. A plausible reaction mechanism was proposed (Scheme 45b). The process was scaled up to gram quantities.

According to Scheme 45b [86], the first step involves the dissolution of hexahydro-1,3,5-triazine **145** to form formalimines **A**, which then further decompose to the corresponding amine **B** and formaldehyde **C** due to the presence of a small amount of water in the solvent. Then, the in situ-formed formalimines **A** undergo a formal azaene-type reaction with *N*-sulfonylhydrazones to give intermediate **D**, which can be isomerized to **E**. Finally, intermediate **E** reacts with in situ-formed formaldehyde **C** to produce the final product **147**. Scheme 45b is proposed based on the results of the reaction product analysis and literature sources.

**Scheme 46:**
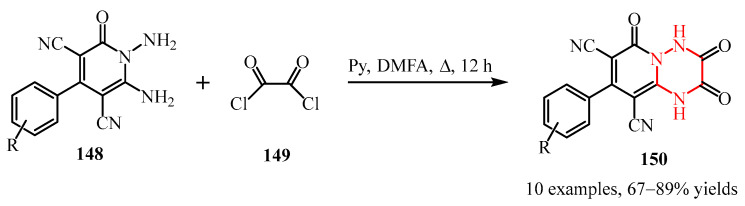
Synthesis of 2,3,6-trioxo-8-aryl-1,3,4,6-tetrahydro-]2*H*[pyrido]1,2-*b*] [1,2,4[triazin-7,9-dicarbonitryls, according to [87]. R = H, Me, OMe, Cl, NO_2_, N(CH_3_)_2_, F, Br, OH.

Substituted triazines **150** were formed by the reaction of 1,6-diamino-2-oxo-4-aryl-1,2-dihydropyridine-3,5-dicarbonitriles **148** with oxalyl chloride **149** (Scheme 46) [87].

#### 3.2.3. 1,3,5-Triazines

**Scheme 47:**
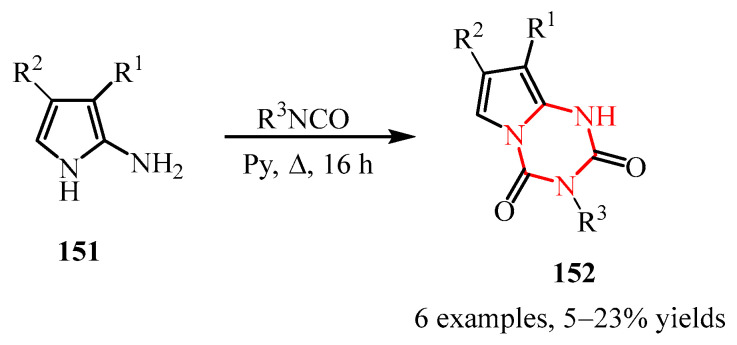
Synthesis of pyrrolo [1,2-*a*][1,3,5]triazine-2,4(1*H*,3*H*)-diones, according to [88].

Treatment of 2-aminopyrroles **151** with aryl- or alkylisocyanates was demonstrated to form 3,7,8-trisubstituted pyrrolo[1,2-*a*][1,3,5]triazindiones **152** (Scheme 47) [88].

**Scheme 48:**
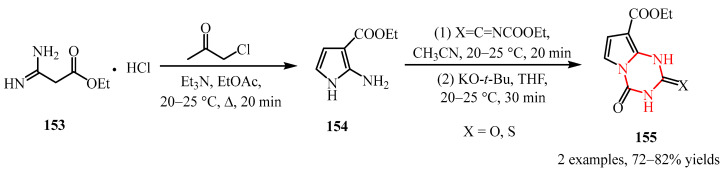
Synthesis of pyrrolo[1,3,5]triazine, according to [89].

Pyrrolo[1,2-*a*][1,3,5]triazines **155** were obtained by a two-step reaction. Firstly, 2-amino-3-ethoxycarbonylpyrrole **154** was synthesized by the Hantzsch method via the condensation of amidine ester **153** and freshly distilled chloroacetaldehyde. Secondly, the reaction of pyrrole **154** with cyanate occurred (Scheme 48) [89].

**Scheme 49:**
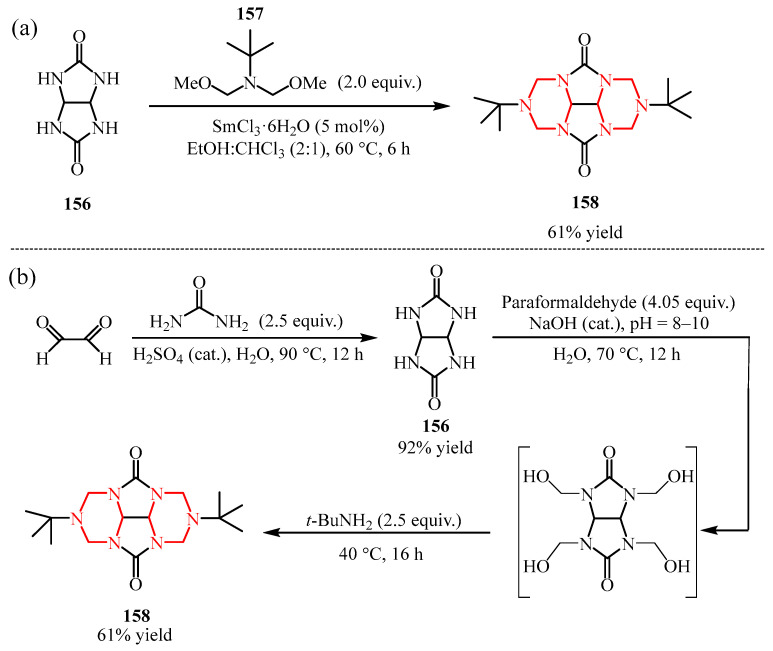
(**a**) Sm-mediated synthesis of di-*tert*-butyl-*bis*-1,3,5-triazinane; (**b**) gram-scale synthesis of *bis*-1,3,5-triazinanes **158**, according to [90].

Sabatini et al. [90] developed an elegant method for the synthesis of *bis*-1,3,5-triazinanes **158** (Scheme 49a). The reaction was carried out in the presence of samarium trichloride as a catalyst, water as a solvent, and glycoluril **157** and *N*,*N*-*bis*(methoxymethyl)-*tert*-butylhexanamine **156** as a substrate. It is interesting to note that the authors of [90] also synthesized compounds **158** on a 176 mmol scale to obtain 36 g of the product in several steps (see Scheme 49b), using paraformaldehyde and *t*-BuNH_2_ instead of the samarium catalyst.

**Scheme 50:**
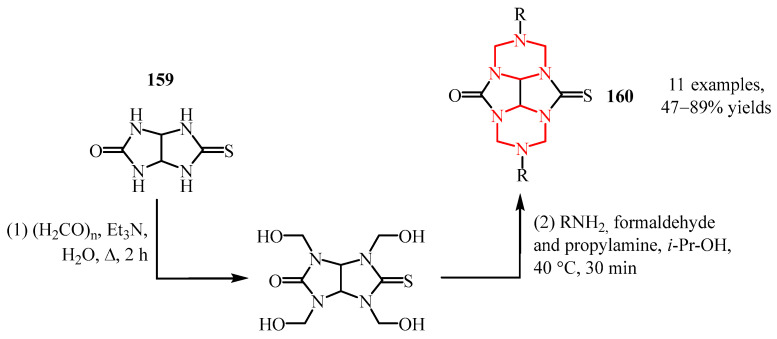
Synthesis of 2,6-disubstituted 2,3a,4a,6,7a,8a-hexaaza-perhydrocyclopenta[*def*]fluorine-4-thioxo-8-ones, according to [19].

Baranov et al. [19] also synthesized *bis*-1,3,5-triazinanes **160** according to (Scheme 50) via the condensation of 5-thioxohexahydroimidazo[4,5-*d*]imidazol-2(1*H*)-one **159** (1, monothioglycoluril) with paraformaldehyde and amines (11 examples).

A series of new fused aza heterocycles containing 1,3,5-triazinane fragments formed from glycoluril, 2,8-*bis*(hydroxymethyl)glycolurils, *tetrakis*(hydroxymethyl)glycoluril, and 1,4,5,8-tetraazadecalin were described in [91,92,93,94].

**Scheme 51:**
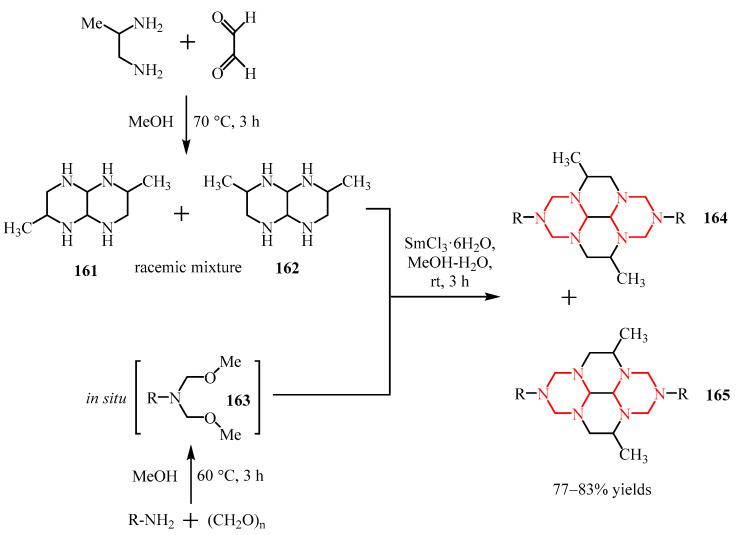
Intermolecular heterocyclization of *N*,*N*-*bis*(methoxymethyl)-*N*-alkylamines with 2,6(7)-dimethyl-1,4,5,8-tetraazadecalins, according to [95]. R = *n*-Pr (81% total yield), *n*-Bu (83% total yield), *i*-Pr (77% yield), and *t*-Bu (79% yield).

The synthesis of 2,7-*bis*-substituted 4,9(10)-dimethyl-2,3a,5a,7,8a,10a-hexaazaperhydropyrenes **164** and **165** (14 examples) via the intermolecular heterocyclization of *N*,*N*-*bis*(methoxymethyl)-*N*-alkylamines **163** with 2,6(7)-dimethyl-1,4,5,8-tetraazadecalines **161** and **162** under rather mild conditions in the presence of samarium catalyst was implemented in [95] (Scheme 51).

**Scheme 52:**
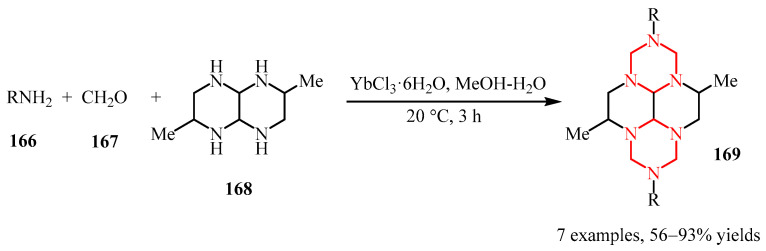
Yb-catalyzed synthesis of 2,7-diaryl(hetaryl)-4,9-dimethyl-perhydro-2,3a,5a,7,8a,10a-hexaazapyrenes, according to [96]. R = 2-HOC_6_H_4_; 4-HOC_6_H_4_; 4-HOC(O)C_6_H_4_; 3-HO-4-HOC(O)C_6_H_3_; 5-methyl-1,2-oxazol-3-yl; 1,5-dimethyl-3-oxo-2-phenyl-2,3-dihydro-1*H*-pyrazol-4-yl; and pyridine-2-yl.

A research team developed a method for the cyclocondensation of arene(hetarene)amines **166** with formaldehyde **167** and 2,6-dimethyl-1,4,5,8-tetraazadecalin **168** in the presence of YbCl_3_·6H_2_O as a catalyst [96] (Scheme 52).

It should be underlined that the products described in [95,96] simultaneously contain piperazine and 1,3,5-triazinane moieties. In addition, all of the methods described by the authors are implemented in a one-pot manner under fairly mild conditions. The only significant drawback is the use of d- or f-element compounds as catalysts.

### 3.3. Tetrazine Compounds

#### 3.3.1. 1,2,3,4-Tetrazine

**Scheme 53:**
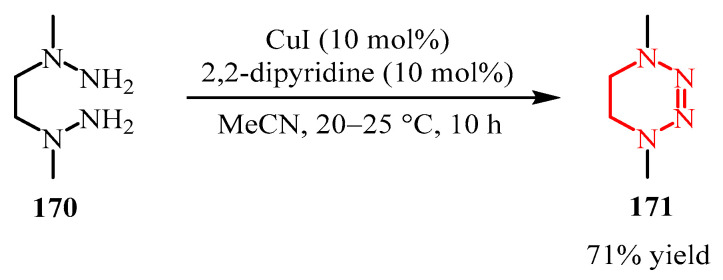
Cu-catalyzed synthesis of cyclic tetrazene, according to [98].

In 2022, *Renault* et al. synthesized 1,2,3,4-tetrazine **171** (Scheme 53) by intramolecular cyclization of hydrazine **170** using CuI as a catalyst [97,98].

#### 3.3.2. 1,2,3,5-Tetrazine

**Scheme 54:**
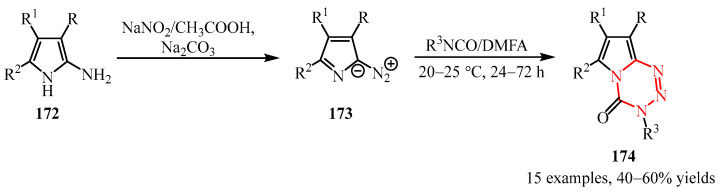
Synthesis of pyrrolo [2,1-*d*][1,2,3,5]tetrazine-4(3*H*)-ones, according to [99]. R = CN, CONH_2_, CO_2_Et; R^1^ = Me, Ph; R^2^ = Me, Ph; and R^3^ = Me, cyclohexyl, Ph, heteroaryl.

Diazoazoles **173** obtained via the diazotization reaction followed by the neutralization of the corresponding pyrroloamines **172** and their further interaction with alkyl- or arylisocyanate furnished pyrrolo[1,2,3,5]tetrazinones **174** (Scheme 54) [99].

#### 3.3.3. 1,2,4,5-Tetrazine

**Scheme 55:**
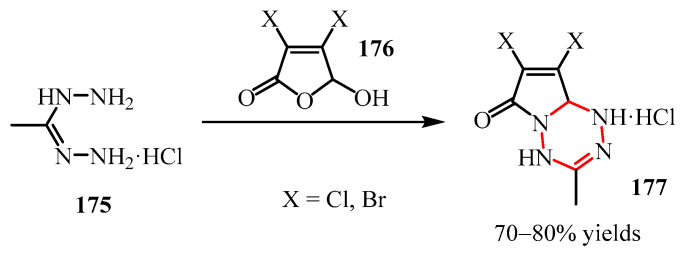
Synthesis of pyrrolo [1,2*-b*][1,2,4,5]tetrazine derivatives, according to [100,101].

An interesting example of the synthesis of pyrrolo[1,2*-b*][1,2,4,5]tetrazine derivatives **177** in good yields was described in [100,101] (Scheme 55). The reaction proceeds through the cyclization of hydrazidine hydrochloride **175** with halogen-substituted 5-hydroxyfuran-2(5*H*)-ones **176**.

**Scheme 56:**
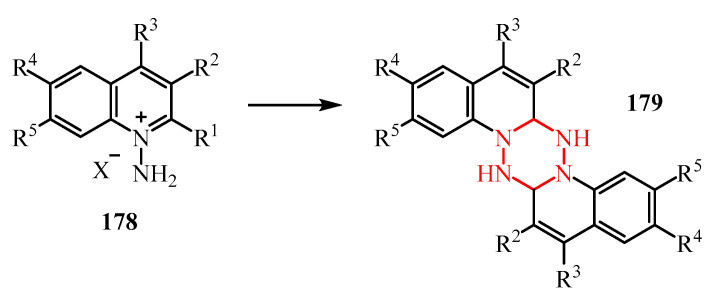
Dimerization of *N*-aminopyridinium salts, according to [102,103]. R^1^ = COR’; R’ = OEt, ClC_6_H_4_; R^2^ = H, Me; R^3^ = H, Me; R^4^ = H, Me, OMe; R^5^ = H, Me, OMe, Cl; and X = OMeS.

The dimerization of compounds bearing the *N*-aminopyridinium salts fragment **178** was reported (Scheme 56). These processes can take place in a basic medium [102,103].

**Scheme 57:**
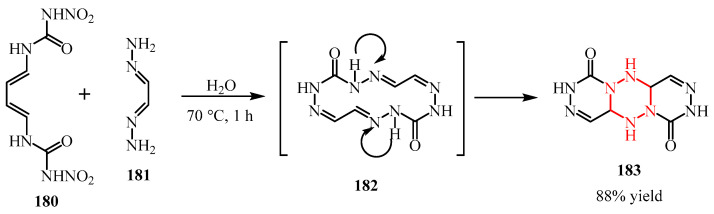
Synthesis of 2,3,4a,6,7,8a,9,10-octaaza-4,8-dioxo-3,4,4a,7,8,8a,9,9a,10,10a-decahydro-anthracene, according to [104].

Another method for the preparation of heteroanthracene derivatives **183** is the interaction of glyoxal-*bis*(hydrazone) **181** with glyoxal-*bis*(nitrosemicarbazone) **180** in water (Scheme 57) [104].

**Scheme 58:**
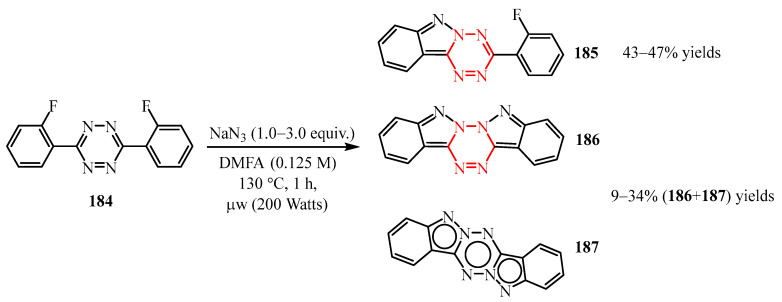
Synthesis of tetrazo [1,2-*b*]indazoles, according to [105].

An interesting example of partial dearomatization/aromatization that results in the formation of tetrazo[1,2-*b*]indazoles **185**–**187** was disclosed in [105] (Scheme 58). These structures exhibited low reduction potential, absorption in the visible (up to the near infrared) region, and intense fluorescence (in the case of *bis*-tetrazo[1,2-*b*]indazole (**184**)). It is noteworthy that both compounds **185**–**187**, in addition to their intrigue from a synthetic perspective, can be regarded as organic semiconductors, thus finding application in a diverse array of photonic and optoelectronic domains.

**Scheme 59:**
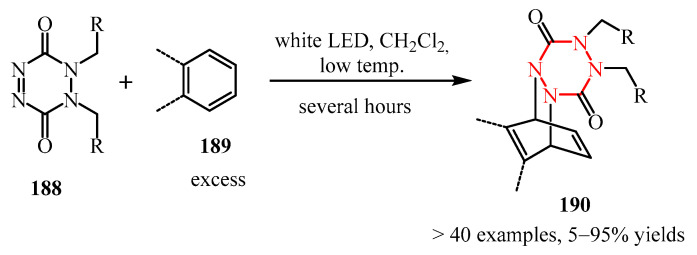
Dearomative [4+2] cycloaddition between 1,2-dihydro-1,2,4,5-tetrazine-3,6-diones and benzenes, naphthalenes, or *N*-heteroaromatic compounds, according to [106]. R = Me, Bn, 2,4-(CF_3_)_2_-C_6_H_3_; **189** = benzenes, naphthalenes, *N*-heteroarenes.

Another remarkable example of a strategy for the dearomatization of tetrazine derivatives is dearomatic [4+2]-cycloaddition of 1,2-dihydro-1,2,4,5-tetrazine-3,6-diones **188** to benzenes, naphthalenes, or *N*-heteroaromatic compounds **189** under visible light irradiation [106] (Scheme 59). In addition to a detailed description of the synthetic procedures, computational studies on retro-cycloaddition reactions were performed.

## 4. Conclusions

The literature analysis presented here is (1) a logical continuation of our previous review; (2) cannot be considered exhaustive; and (3) indicates that the preparation of saturated or partially saturated polyazocyclic skeletons lacking aromaticity is one of the most challenging problems in modern organic synthesis and catalysis. The three general strategies described in the literature, namely hydrogenation (hydrogen addition) and saturation (addition of molecules other than H_2_, e.g., hydroboration) of six-membered polyazo heterocycles, as well as construction of polyazocyclic compounds (including that via binding of two or more molecules, ring expansion reactions, and intramolecular and intermolecular cyclizations), have their own advantages and shortcomings. For instance, the process of classical hydrogenation, which is carried out in the presence of molecular hydrogen and a catalyst, necessitates elevated hydrogen pressure. In order to obtain chiral products, either a chiral catalyst or an additional chiral ligand should be employed, which must remain chiral throughout the entire process that can sometimes last for many hours. Saturation via hydroboration leads to the introduction of additional functional groups into the cyclic polyazoate moieties, which are not always desirable. Consequently, an additional reaction step in which these functional groups are substituted may be required. Despite their apparent simplicity, construction processes of the polyazole core through binding of two or more molecules, ring expansion reactions, as well as intramolecular and intermolecular cyclizations, contribute not only to the partial or complete saturation/formation of polyazo nonaromatic rings, but also lead to the introduction of additional functionalization into the target skeletons and require the use of catalysts of varying degrees of complexity, etc. The resolution of the aforementioned issues has been instrumental in propelling this research field forward, particularly in the last decade. However, the predominant focus of scientific groups on individual examples has resulted in an augmentation of intriguing techniques from a synthetic perspective. Nevertheless, this approach has not contributed to the systematic accumulation of results necessary for the formulation of a comprehensive theory, at least at this stage of research.

## Data Availability

No new data were created or analyzed in this study. Data sharing is not applicable to this article.

## Abbreviations

The following abbreviations are used in this manuscript:

ACN	Acetonitrile
AcOH	Acetic acid
anti-HIV-1	Active against human immunodeficiency virus
CbzCl	Benzyl chloroformate
cod	1,5-Cyclooctadiene
DCE	1,2-Dichloroethane
DCM	Dichloromethane
DCC	1,3-Dicyclohexylcarbodiimine
dF(CF_3_)ppy	2-(2,4-difluorophenyl)-5-(trifluoromethyl)pyridine
DIPEA	*N*,*N*-Diisopropylethylamine
DMA	*N*,*N*-Dimethylaniline
DMFA	Dimethylformamide
dtbpy	4,4′-Di-tert-butyl-2,2′-bipyridine)
*ee*	Enantiomeric excess
er	Enantiomeric ratio
EtOAc	Ethyl acetate
HAT	Hydrogen-atom transfer
HBpin	Pinacolborane
HRMS	High-resolution mass spectrometry
ISC	Intersystem crossing
LED	Light-emitting diode
LOHC	Liquid organic hydrogen carriers
MeOCOCl	Methyl chloroformate
MTBE	Methyl tert-butyl ether
MW	Microwave
NMP	N-Methyl-2-pyrrolidone
OTf	Triflate group
PMB	4-Methoxybenzyl
Py	Pyridine
(*R*)-Segphos	(*R*)-(+)-5,5′-*Bis*(diphenylphosphino)-4,4′-bi-1,3-benzodioxole, [4(*R*)-(4,4′-Bi-1,3-benzodioxole)-5,5′-diyl]*bis*[diphenylphosphine]
(*R*,*S*)-PPF-P-*t*-Bu_2_	(*R*)-1-[(*S*_P_)-2-(Diphenylphosphino)ferrocenyl]ethyldi-*tert*-butylphosphine
(*R Sp*)-Josiphos	{(*R*)-1-[(Sp)-2-(Dicyclohexylphosphino)ferrocenyl]ethyldi-*tert*-butylphosphine}[2-(2′-amino-1,1′-biphenyl)]palladium(II) methanesulfonate
(*S*,*S*)-f-Binaphane	1,1′-*Bis*{(*S*)-4,5-dihydro-3*H*-binaphtho [1,2-*c*:2′,1′-*e*]phosphino}-ferrocene
(*S*)-BINAP	(*S*)-(−)-2,2′-Bis(diphenylphosphino)-1,1′-binaphthalene
(*S*)-tol-BINAP	(*S*)-(−)-2,2′-*p*-Tolyl-phosphino)-1,1′-binaphthyl, (*S*)-Tol-BINAP
SET	Single-electron transfer
(*S*)-Segphos	(*S*)-(−)-5,5′-*Bis*(diphenylphosphino)-4,4′-bi-1,3-benzodioxole
(*S*)-Synphos	6,6′-*Bis*(diphenylphosphino)-2,2′,3,3′-tetrahydro-5,5′-bibenzo[*b*][1,4]dioxine
(*S*)-DM-Segphos	(*S*)-(−)-5,5′-*Bis*(diphenylphosphino)-4,4′-bi-1,3-benzodioxole
(*S*)-DTBM-Segphos	(*S*)-(+)-5,5′-*Bis*[di(3,5-di-*tert*-butyl-4-methoxyphenyl)phosphino]-4,4′-bi-1,3-benzodioxole
(*S*)-Difluorphos	(2,2,2′,2′-Tetrafluoro-4,4′-bibenzo[*d*][1,3]dioxole-5,5′-diyl)*bis*(diphenylphosphine)
TBAB	Tetrabutylammonium bromide
temp	Temperature
TFA	Trifluoroacetic acid
Troc	2,2,2-Trichloroethoxycarbonyl
TrocCl	2,2,2-Trichloroethoxycarbonyl chloride
Xantphos	(9,9-Dimethyl-9*H*-xanthene-4,5-diyl)*bis*(diphenylphosphane)
US	Ultrasound
Δ	Boiling or reflux
*	Optically active fragment or center
